# A systematic review of sub-national food insecurity research in South Africa: Missed opportunities for policy insights

**DOI:** 10.1371/journal.pone.0182399

**Published:** 2017-08-22

**Authors:** Alison Misselhorn, Sheryl L. Hendriks

**Affiliations:** 1 Institute for Food, Nutrition and Well-being, University of Pretoria, Pretoria, Gauteng, South Africa; 2 Institute for Food, Nutrition and Well-being and the Department of Agricultural Economics, Extension and Rural Development, University of Pretoria, Pretoria, Gauteng, South Africa; Institut de recherche pour le developpement, FRANCE

## Abstract

Food insecurity is an intractable problem in South Africa. The country has a tradition of evidence-based decision making, grounded in the findings of national surveys. However, the rich insights from sub-national surveys remain a largely untapped resource for understandings of the contextual experience of food insecurity. A web-based search identified 169 sub-national food insecurity studies conducted in the post-apartheid period between 1994 and 2014. The systematic review found that the studies used 27 different measures of food insecurity, confounding the comparative analysis of food insecurity at this level. While social grants have brought a measure of poverty relief at household level, unaffordable diets were the root cause of food insecurity. The increasing consumption of cheaper, more available and preferred ‘globalised’ foods with high energy content and low nutritional value lead to overweight and obesity alongside child stunting. Unless a comparable set of indicators is used in such surveys, they are not able to provide comparable information on the scope and scale of the problem. Policy makers should be engaging with researchers to learn from these studies, while researchers need to share this wealth of sub-national study findings with government to strengthen food security planning, monitoring, and evaluation at all levels.

## Introduction

It is well documented that food insecurity is caused by structural inequalities [1]. Since 1994, food security has been acknowledged as a national priority [2]. This prioritisation is evident in the key guiding national policies such as the Reconstruction and Development Programme (1994) [3], the Integrated Food Security Strategy (2012) [4] and later the National Policy on Food and Nutrition Security [5]. Over the last two decades following South Africa’s transition to a democratic state, food security has received significant policy attention and a range of interventions have been implemented by the Government, NGOs, civil society groups and the public sector [6].

However, South Africa has no official measure of food insecurity although the 2014 National Policy on Food and Nutrition Security’s [5] implementation plan includes a list of possible indicators, the measures, these have not been formally adopted as official measures for regular reporting across monitoring and evaluation systems in various national departments and bodies. Regular national surveys report on various elements of food access, the experience of hunger and nutrition statistics [7]. It is reported that around 26% of South African households or 13–15 million people have either inadequate or severely inadequate access to food [7]. Recently, Africa Check [8] have queried the validity of such claims. Africa Check explains that: “Determining how many hungry people there are in South Africa is not as straightforward as calculating 26% of the population”. Hendriks et al. (2016) have noted that the rich body of sub-national research studies is largely ignored and could provide important information regarding experiences of individuals, households, and communities to assist in determining the scope and scale of hunger, malnutrition and food insecurity in the country [9].

This paper presents the findings of a systematic review that set out on an ambitious task to explore and document the reality of food insecurity in South Africa over 20 years from the 1994 dawn of democracy to 2014 by drawing on the large body of hitherto un-synthesised peer-reviewed, published and unpublished research. The review sought to answer two research questions. Firstly, how is food insecurity experienced in South Africa, including the challenges to food access, availability, utilisation (nutrition) and stability and the short and long-term consequences (including coping, adapting, trade-offs) of food insecurity? Secondly, the study explored the implications of the above for decision makers and policy makers at all scales in South Africa.

## Methods

This study adopted a structured systematic review process. A brief review was undertaken of the theory and practice of systematic reviews relevant to social science research [10–19]. More specific review methods were then considered, including meta-synthesis [20], thematic synthesis [21], narrative reviews [22–24], realistic reviews [25–27], and framework analysis [28–32], as well as existing systematic reviews related to food security [33–43]. This exercise informed the research design and theoretical framework.

A preliminary search of the food security literature was undertaken using through the University of X’s online databases, including Sabinet, Academic Search Complete via Ebsco Host, and SCOPUS using the following search terms:

- “Food security” AND “coping” AND “south Africa
- “Food” AND “coping” AND “South Africa”
- “Food security” AND “study” AND “South Africa”.

**Fig 1:**
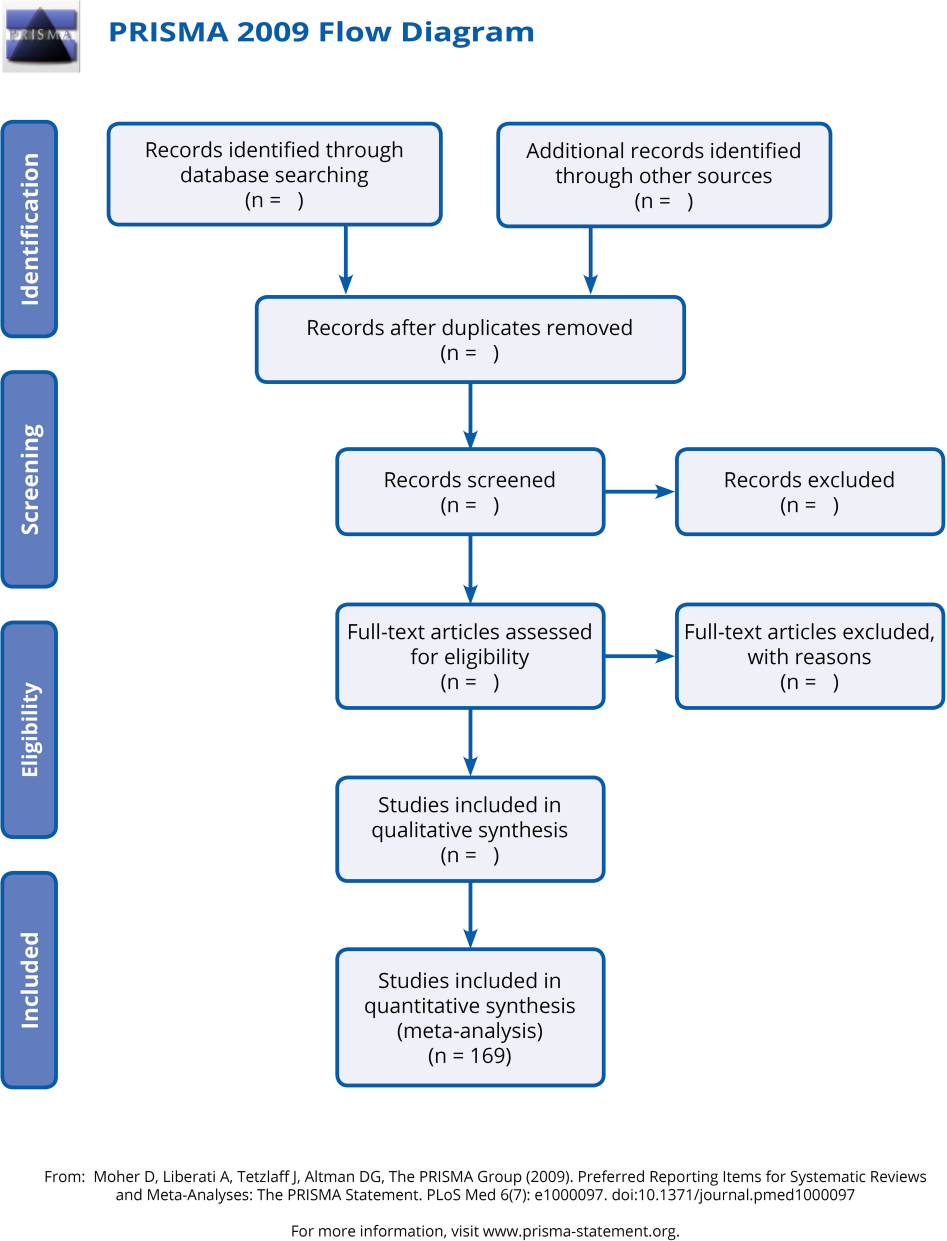
PRISMA flow diagram.

**Fig 2:**
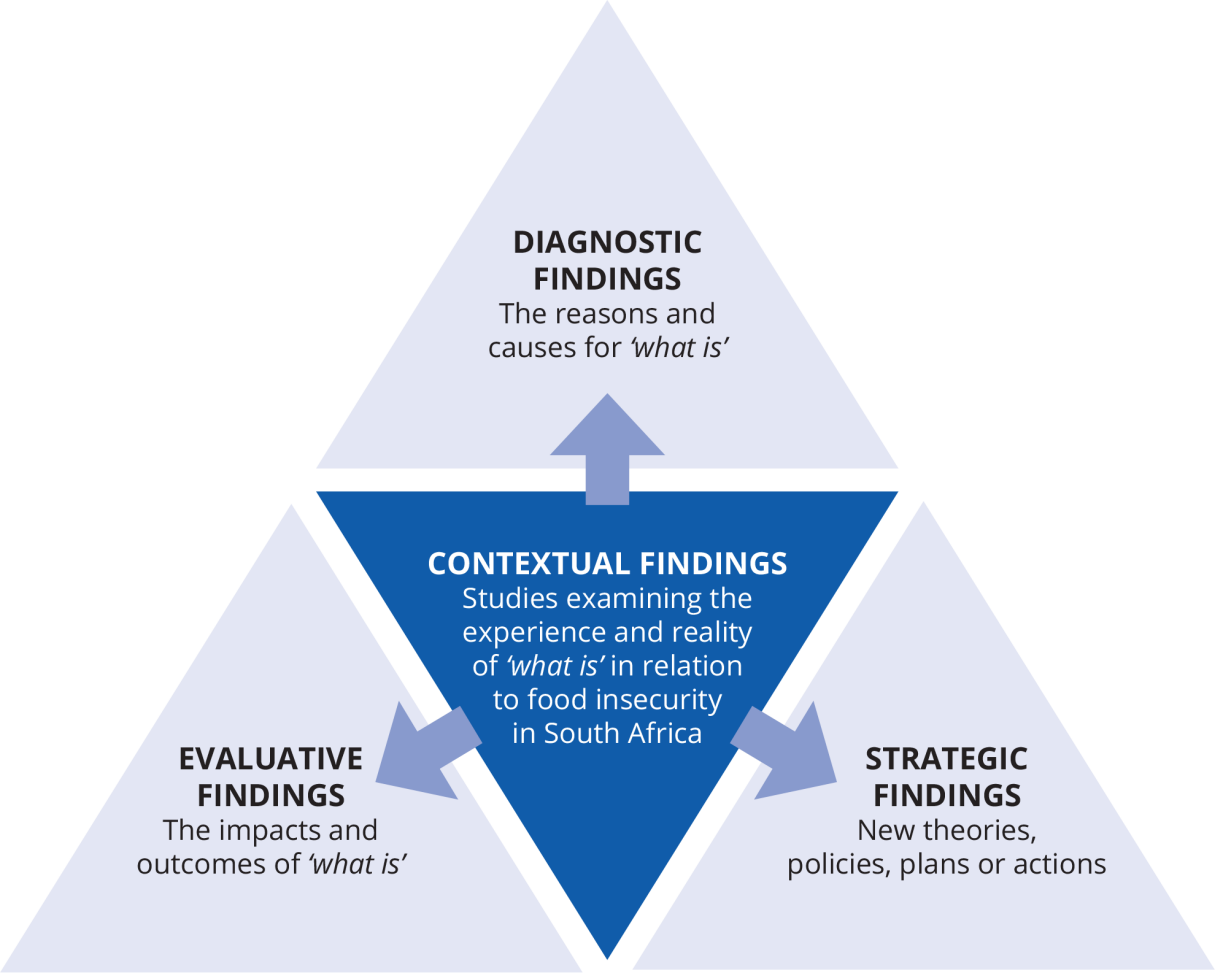
The four categories in the theoretical framework for the study.

A coding (theoretical) framework was developed, based on applied policy research broadly falling into four categories: contextual, diagnostic, evaluative and strategic [18]. Fig 1 indicates the flow diagram for record identification, while Fig 2 presents a diagrammatic representation of the four categories. *Contextual findings* referred to the primary category in that the other three were dependent on these findings. Contextual findings related to the question of *the ‘state’ of food insecurity in South Africa* and how food insecurity was experienced, as evidenced in the studies reviewed. Sub-categories encompassed measurable outcomes of food insecurity, such as malnutrition, as well as attitudes, perceptions, and needs. The remaining three categories were ‘secondary' in that they drew on the findings from the ‘primary' category. The first of these, *diagnostic findings*, speak to the *causes or reasons* for the state of food security and relate to failures in the principle determinants of food security. These included: food stability (variability over time of supply and access), food access (mediating factors of affordability, allocation, power relations), food utilisation (nutritional value in terms of dietary quality, diversity and quantity, social value, food preparation and safety); and food availability (production, distribution and exchange) [44, 45]. The nutritional dimension is integral to the concept of food security.

*Evaluative findings* related to the *impacts and outcomes* of food insecurity. Prior to data analysis, these were defined very broadly to fall under the five key resources people can draw on in their lives and livelihoods to survive, secure food and pursue their wellbeing namely: human, social, financial, physical and natural capitals. Finally, the *strategic findings* related to new theories, policies, responses, plans and actions among all stakeholders, and required drawing on literature and documentation additional to the studies included in the review.

The criteria for inclusion and exclusion of a study and the final set of keywords and search terms were finalised following a peer review by two independent reviewers who evaluated the methods and the coding framework proposed. The final set of inclusion and exclusion criteria were that each study needed to:

- Have been written or published between the start of 1995 and the end of 2014 (20 years)
- Focus on one or more aspects of the state or ‘lived’ experience of food insecurity (access, utilisation, availability of food) or one or more aspects of nutrition (macro and micro-nutrition malnutrition, obesity)
- Be primary empirical work undertaken at sub-national scale
- Have aims and objectives clearly stated
- Have a clear description of methods used, including data collection, sampling and analysis, and
- Show attempts to determine the reliability or validity of the data analysis.

The terms used in the search are presented in Table 1. These terms were employed in searching the University of X’s online electronic databases, specialist websites (including local and global NGOs such as IFPRI, WHO, and others), and eliciting additional studies through personal contact with authors and experts in the field. The initial body of literature included 220 studies. The research study details were recorded in MS Excel in the *apriori* descriptive mapping categories of geographic focus, aims of the study, peer review status, type of report, key findings, methods and sample size. Inclusion and exclusion criteria were applied to the title, abstracts and keywords. An *Atlas*.*ti* project was developed to simultaneously filter and code the studies. Finally, a body of 169 studies was included in the *Atlas*.*ti* database. The full list of studies is provided in S1 Appendix, which also shows the primary groups each document was assigned to.

**Table 1:** List of search terms.

“South Africa” AND (“Food Security” OR “Nutrition”) AND:		
“Study”	“Bush meat”	“Food choices”
“Hung*”	“Wild food”	“Coping”
“Malnutrition”	“Traditional food”	“Adapting”
“Obesity”	“Food storage”	“Trade-offs”
“Food quality”	“Cooking”	“Climate change”
“Food safety”	“Preparation” “Allocation”	“Climate variability”
“Food production”	“Consumption”	“HIV”
“Food insecure*”	“Intra-household allocation	“AIDS”
“Agriculture”	“Gender”	“Food garden”
	“Food preferences”	“Home garden”
		“Urban”

## Results and discussion

**Fig 3:**
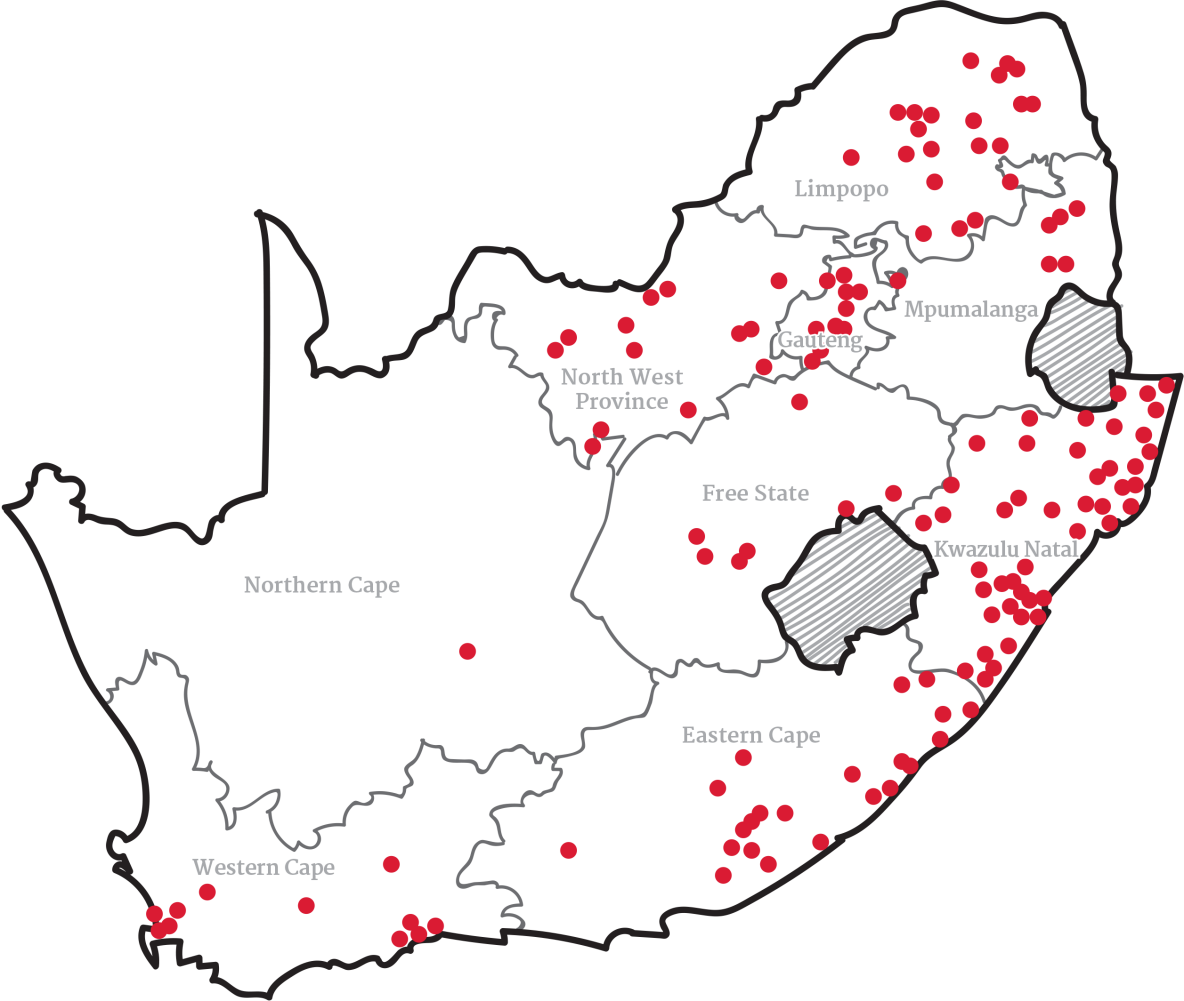
Map indication location of the reviewed studies.

**Fig 4:**
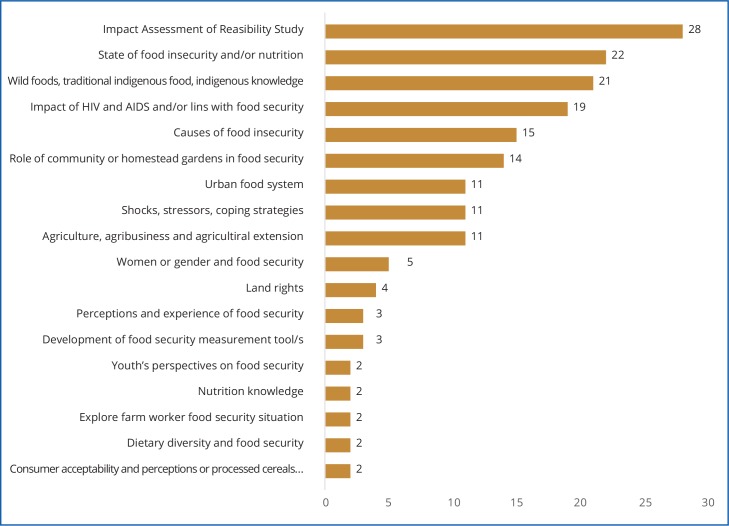
The range of study objectives.

The aims and disciplinary foci, socio-economic and environmental contexts, as well as methods, varied considerably across the studies. Each of the 169 studies in this review focused on different geographic environments and unique socio-economic contexts (Fig 3). A large proportion of studies (107) focused on rural communities, but even within these areas, an enormous diversity was evident. Similarly, the aims and objectives of the studies were diverse, which means their findings focussed on an array of different issues related to food security. The categorised range of the aims and objectives of the studies is shown in Fig 4. There are also many different ways in which the researchers of the included studies attempted to measure food insecurity, with the choice of measurement tool likely being at least partially influenced by the study context and aims.

**Fig 5:**
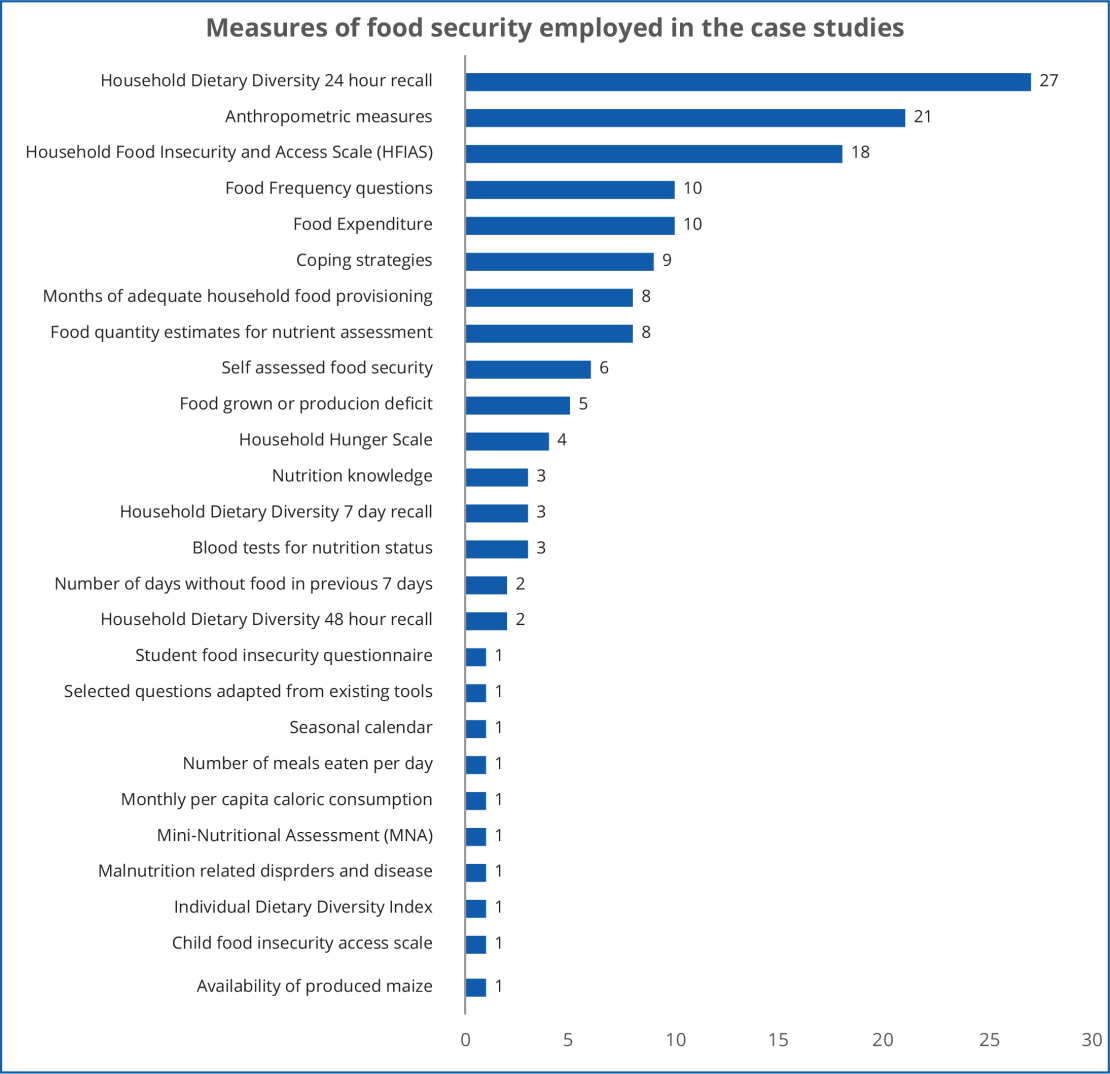
Measures of food insecurity.

The multiple tools and methods used to gauge food security status across the 169 studies in this review are shown in Fig 5. If an author used more than one measure, both were counted. Thirty-four of the studies did not directly measure food security or did not reveal what measure(s) they used.

Some measures, such as anthropometry (to assess under-nutrition), or the coping strategies index, essentially gauge the impacts or symptoms of food insecurity. Others are very narrow in their interpretation of determinants of food security–such as those only assessing the amount maize a household grows. The measures of food security depicted in Fig 5 can be broadly grouped into three approaches to assessing food insecurity, namely those that focus on:

- Food security rather than nutrition—they attempt to establish a ‘level’ of food insecurity, often related to food quantity and/or diversity;
- Nutritional aspects of food security—macro- and micro-nutrient quantity and/or quality of intake; and
- Anthropometric outcomes of poor nutrition using anthropometric measures.

Although these ‘categories’ are not mutually exclusive, their different focus is evident in the language of the 192 coded quotations across the studies that described the ‘state’ of food security among their participants. If an author used more than one measure, both were counted. These quotations were explored in *Atlas*.*ti*, and grouped under the three categories above. See S1 Appendix for more detailed network diagram for these.

The range of food security ‘levels’ described varies enormously across the studies. This is likely to reflect the varying definitions and tools, as well as the situation-specific experiences. Irrespective of how it is measured, the studies confirm that the ability to secure food remains an uncertainty for the majority of South Africans. Even if only studies with sample sizes over 200 are included from the general population (excluding those covering ‘special populations' such as HIV-positive individuals), the picture is bleak (Table 2). An average of 68% of participants in these studies experiences difficulty in securing a dependable supply of food. The figures are not at odds with those evident in National level surveys, with the percentage of households in the country that ‘run out of money to buy food' estimated at 70% in the National Food Consumption Survey of 2005, and 46% in the South African Social Attitudes Survey of 2008 [46].

**Table 2:** Food Security estimates in studies examining levels of food security in the general population with sample sizes over 200.

Proportion of food insecure in the sample	Location
53% severely food insecure	Rural Limpopo (Study 144) [47]
Nearly 90% food insecure	Urban KwaZulu-Natal (Study 88) [48]
73% rural and 87% urban families at high risk of food insecurity	Urban and Rural Free State (Study 1) [49]
76% worry about not enough money for food	Rural Limpopo (Study 82) [50]
69% of households severely food insecure	Rural Eastern Cape (Study 76) [51]
64% of female and 42% of male headed households food insecure	Peri-Urban Free State (Study 50) [52]

Impacts on growth and physical development are among the many impacts of food insecurity experienced by South Africans. Stunting–having a height for age below minus two standard deviations below the population norms—ranged from 16% to 48% in the various studies, but the studies are all unique in their sample type (including age groups) and focus (Table 3).

**Table 3:** Estimates of retarded physical development in those studies which measured weight for height or height and weight for age.

Proportion of the sample	Location
48% of children (35–37 months) were stunted	Rural Limpopo (Study 98) [53]
19% of children (36–79 months) were stunted	Urban North West (Study 132) [54]
29% of children (6–72 months)were severely wasted	Urban and Rural KwaZulu-Natal (Study 15) [55]
25% of girls (36–47 months) were underweight	Peri-Urban Gauteng (Study 29) [56]
35% of children (6–71 months) were stunted, 11% underweight	Rural Limpopo (Study 151) [57]
19% of learners (10–12 yrs) were stunted	Rural North West (Study 141) [58]
44% of children (1–16 yrs) were either underweight or stunted	Rural North West (Study 115) [59]
18% children (1–4 yrs) were stunted, 32% of 1 year olds	Rural Mpumalanga (Study 30) [60]
32% of pupils (10–12 yrs) had low weight and height for age	Rural North West Province (Study 139) [61]

The National Food Consumption Surveys of 1999 and 2005 suggest that stunting in South African children has hardly decreased from 21,6% in 1999 to 18% in 2005 [46]. However, these figures still mask the significant inter-community and inter-household variations in nutrition security in which some are mired in chronic, debilitating insecurity marked by retarded development, and others are far less affected.

Some studies included in this review make the argument that the evidence that food insecurity–or even inadequate dietary intake–causes stunting is weak. In a study in Gauteng and Limpopo Provinces, a quantitative food-frequency questionnaire was used to assess the diets of 30 stunted children in a rural area and 40 in an urban area through interviews with their mothers/caregivers (study 149) [62]. Diets of inadequate quality were found in both groups with no significant differences. The authors of this study conclude that inadequate dietary quality was not the primary cause of stunting. An earlier study, in the North West Province, measuring weight and height for age among 396 children 10–12 years found that differences in diet did not influence whether a child was below or above the 5^th^ centile ([61] pg 95). The authors conclude that: “*there must be caution in over blaming under nutrition*, *and overrating the health disadvantages from mild to moderate malnutrition*” (Study 139) ([61] pg 9). A study in the rural Free State province found no correlation between dietary diversity and BMI; though the reasons are not explored (Study 73) [63].

While these studies do not explore possible impacts of nutritional deficits other than stunting, they do raise important questions about how food security is measured.

## Challenges to food access, food utilisation, food availability, and food nutrition

**Fig 6:**
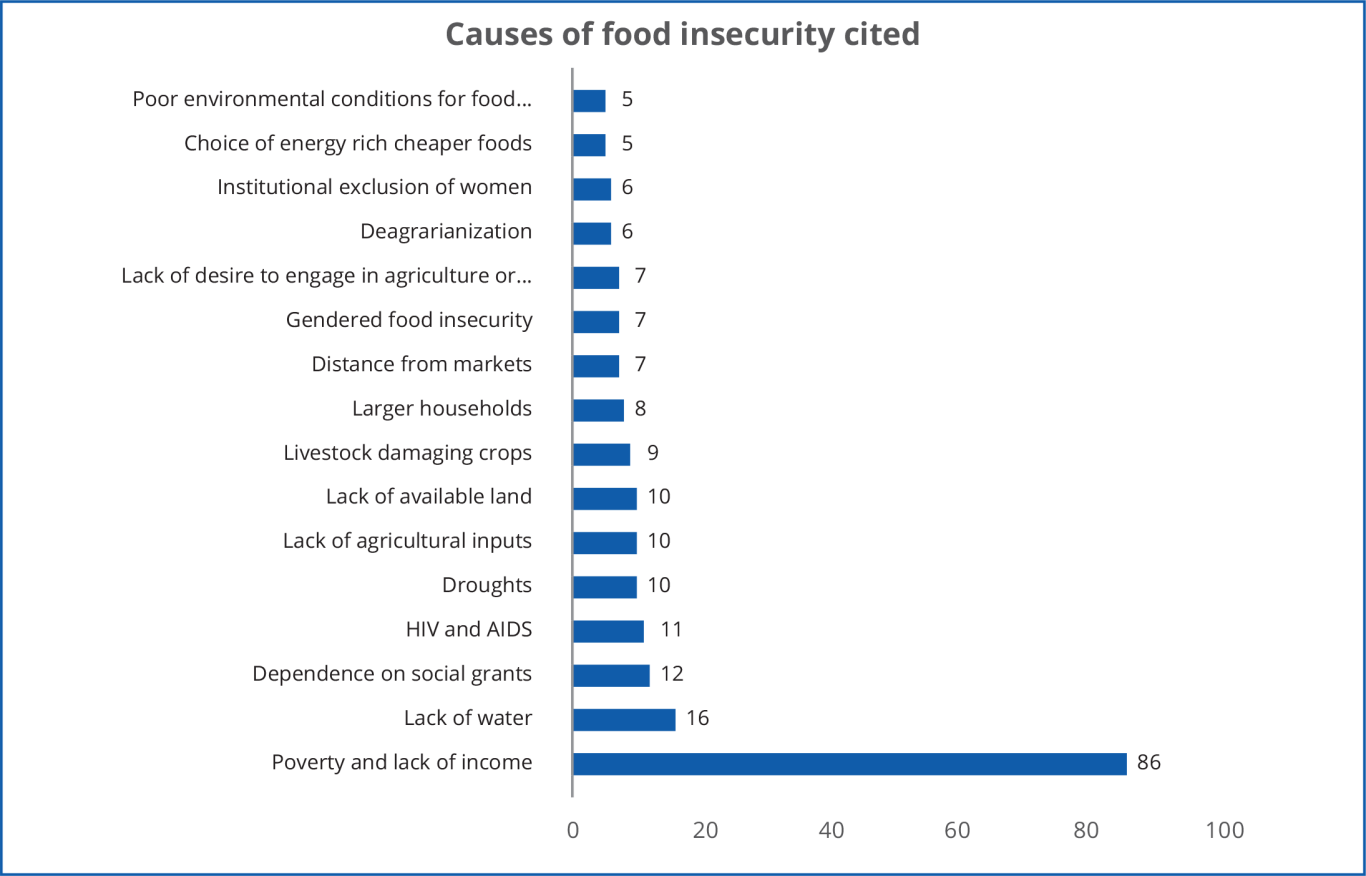
The causes of food insecurity cited in the 169 studies in the review.

The causes and consequences of food insecurity in South Africa are not uniform from place to place and from household to household. There were over 90 discrete codes assigned in *Atlas*.*ti* to causes of food insecurity cited in the 169 studies. Fig 6 shows only those causes cited in five or more studies, indicating to some extent the range of social, economic and biophysical dynamics of food insecurity in the country. The following sections discuss key thematic areas that emerged in the *Atlas*.*ti* analysis that also are reflective of how the causes of food insecurity are understood in the studies.

### Poverty

Despite variation and complexity, poverty remains the principal underlying cause of South Africa’s food insecurity. It is by far the most-cited driver, with 51% (86) of the studies in the review citing poverty and/or lack of income as a cause of being food insecure. Of these, 70% (60) studies empirically assessed household or individual income against food security, in 12 participants themselves cited poverty as a cause of their food insecurity, in nine studies participants revealed they often ran out of money to buy food. Four of the studies citing poverty as a driver of food insecurity drew on the author(s)' view from the evidence, and one assessed household assets against food security status.

There are many factors that can help alleviate food insecurity and malnutrition, but being able to afford to purchase food remains a key determinant. Living in poverty also has secondary livelihood impacts that further entrap people in a state of food security. The central role of poverty and unemployment has important implications for interventions and decision-making.

Even rural South Africans are highly reliant on food purchase as a means to access food [64, 65], and rural households in South Africa spend a higher proportion of their total expenditure on food than their urban counterparts, even though their per capita gross expenditure on food is lower [66]. However, it must be kept in mind that higher transaction costs are common for rural households in accessing purchased food, such as the cost of transport to markets.

Food affordability is governed by both food prices and purchasing power (income), which means economic downturns bring a double threat to the food security of low-income households. The food price crisis between 2007 and 2009 highlighted the precarious levels of affordability of food for many South Africans. The impact of past and future food price increases can increase hunger and also decrease dietary quality, with a higher reliance on affordable staple starches and lower consumption of fruit and vegetables [66, 67]. This has multiple consequences for children’s physical, psychological, educational, and social development.

Referring to the 2008 General Household Survey (GHS), Jacobs highlights a rise in the experience of household hunger by two to three percentage points following food price inflation during 2007/8, together with the general economic downturn [68]. Female-headed households were more severely affected, and in terms of location, those most affected were in rural areas of the Eastern Cape and KwaZulu-Natal. Engel’s law [69]–that the share of a household’s expenditure on food increases as poverty increases–is borne out in the 2008 General Household Survey [68]. Households experiencing the worst food insecurity which adults ‘often or always go hungry’ reported a 67% share of expenditure on food [68].

The ability to access and purchase food in markets is a key consideration for both urban and rural food security, which highlights the influential role of food retail in the food system. The food sector in South Africa is increasingly dominated by major retail groups, which has arguable advantages in keeping food prices down due to purchasing power and economies of scale [70]. However, the impact is not quite that straightforward. The dominance of major food retail groups means that it is increasingly difficult for small-scale producers to break into a market that is centralized under corporate, buyer-driven control, and favours large-scale producers [70]. Smaller food outlets (formal and informal) are also increasingly squeezed out due to price competition, yet these traders are often preferred by consumers for their better geographic accessibility, and also represent critical livelihoods for many [70]. In the African Food Security Urban Network (AFSUN) survey of eleven African cities, 2008–2009, about 70% of households sourced food from informal outlets across all the surveyed cities. In Johannesburg this figure was 85% [70].

### The role of government social grants

Given the pivotal role of poverty in food security, it is to be expected that social protection plays a prominent role in food security in South Africa. This is one of the Government’s primary tools in mitigating the impacts of poverty and unemployment, and many of the studies included in the review make reference to the high level of dependence their participants have on social grants for food security (Studies 46, 55, 60, 66, 80, 82, 96, 101, 108, 142, 158) [50, 71–80]. These studies have disparate study aims, ranging from intervention impact assessments to exploring the relationship between culture and food security.

Almost all of the studies emphasize that grants such as the child support grants prevent absolute food insecurity. One study called for an increase of social protectionist policies in the context of HIV and AIDS and food security (Study 60) [73], and another for attention to the removal of barriers to grant access as an important policy consideration (Study 96) [75]. Grants were also argued in studies to contribute to transforming gender relations (Studies 116, 143) [81, 82] (See the section below on gender dynamics).

Those affected by HIV and AIDS are often particularly dependent on grants. In one study undertaken among 82 HIV/AIDS-affected households the Capricorn District in Limpopo Province, about 42% were found to rely on more than one social grant for their survival (Study 66) [80]. The potential double-edged impact of social grants is well illustrated in the nexus between HIV and AIDS and the disability grant. Trade-offs may come into play, suggesting the on-going challenge that Governments face in mitigating short-term deprivation without undermining long-term development. In a study based on 14 months of urban ethnographic field work in the Western Cape with 35 HIV-positive men and women, the links between HIV/AIDS and food security were investigated (Study 91) [83]. Patients were found to be modifying their adherence to ART in order to maintain low CD4 counts so that they would remain eligible for the disability grant; a clear example of a conflict of interests between long-term health and immediate economic survival. Similarly, two studies in the review found that the grant system acted as a disincentive to homestead food farming–or subsistence farming (Studies 55, 158) [72, 79]. In Study 55, a combination of annual survey work, interviews, participant observation, and ethnographic field research work was drawn on to conclude that grants buffered against absolute food insecurity, but also as provided for enough food purchase to discourage homestead food gardening. In Study 158, the claim that social grants, along with factors such as access to cheap sources of alcohol, had reduced the motivation of the men in the community to engage in farming came directly from a study participant.

Taking a food systems perspective, a study investigating the role of the retail sector in food security in the face of declining subsistence agriculture argued that for a food system to be truly adaptive, it cannot be dependent on external social assistance (Study 43) [84]. This work is based on survey data from Mpumalanga Province with 117 households in three villages.

This high dependence on social grants is by no means a new finding, but what is notable is the schism among the studies, in which grants were viewed variously as aides and obstacles to long-term food security.

### Social capital

Social capital is largely a relational human resource; both accrued and built through interaction between people and within and between groups. It is a somewhat contested concept, partly because it does not introduce previously unexplored resources into social science, rather providing a means to group, examine and articulate them. Social capital in relatively recent literature is taken to include relations of trust, reciprocity, and exchange, common rules and norms, which serve to create connectedness–bonding, bridging or linking–between people and groups (See for e.g. [85, 86, 87]). Social capital is relatively intangible and as a result difficult to measure. Moreover, it does not herald universally benign impacts on people’s lives. Sometimes it brings negative consequences, such as exclusion and imbalances of power (See for e.g. [88]).

Nevertheless, social capital has been linked for some time with a range of development benefits (e.g. [89]), such as the ability to manage livelihood shocks [90]. Elements such as trust and social harmony have been found to be positively associated with child nutritional status across four developing countries (Peru, Ethiopia, Vietnam and a state of India) [91]. Drawing on data from the KwaZulu-Natal Income Dynamics Study, households in communities with higher levels of social capital were found to be more likely to cope with economic shocks [92].

Not many studies included in the review incorporated an explicit focus on the role of social capital. What is clear from those that do is the context-specific nature of the social dynamics of food security. Food security policies and programming cannot escape these dynamics if interventions are to have a real impact on the experience of food insecurity in South Africa. A multi-disciplinary five-year study on rainwater harvesting in the Eastern Cape found that social meaning, as well as social networks and relationships, were critical to the success of future rainwater harvesting interventions (Study 55) [72].

A study explicitly focusing on the relationship between social capital and food security was undertaken among 50 households in a peri-urban village in KwaZulu-Natal (Study 90) [93]. This work found that three forms of social capital play a pivotal role in household food security–church membership, social networks and savings club. Their roles, however, played out in very different ways in different households, in which the same community level institution can bring positive impacts for some households but exclusionary impacts for others (Study 90) [93].

Similarly, an investigation into the relationship between food security and attributes of social capital in two villages in Mpumalanga found no relationship between social capital and food security in one village, but significant relationships between food security and collective action and cooperation, social cohesion, and self-esteem in the other (Study 31) [94].

Together, these studies emphasize the mutable and context-specific nature of social resources, which are inescapably critical to lives and livelihoods, but very challenging to consider in policies and programming.

### Role of food gardens

**Fig 7:**
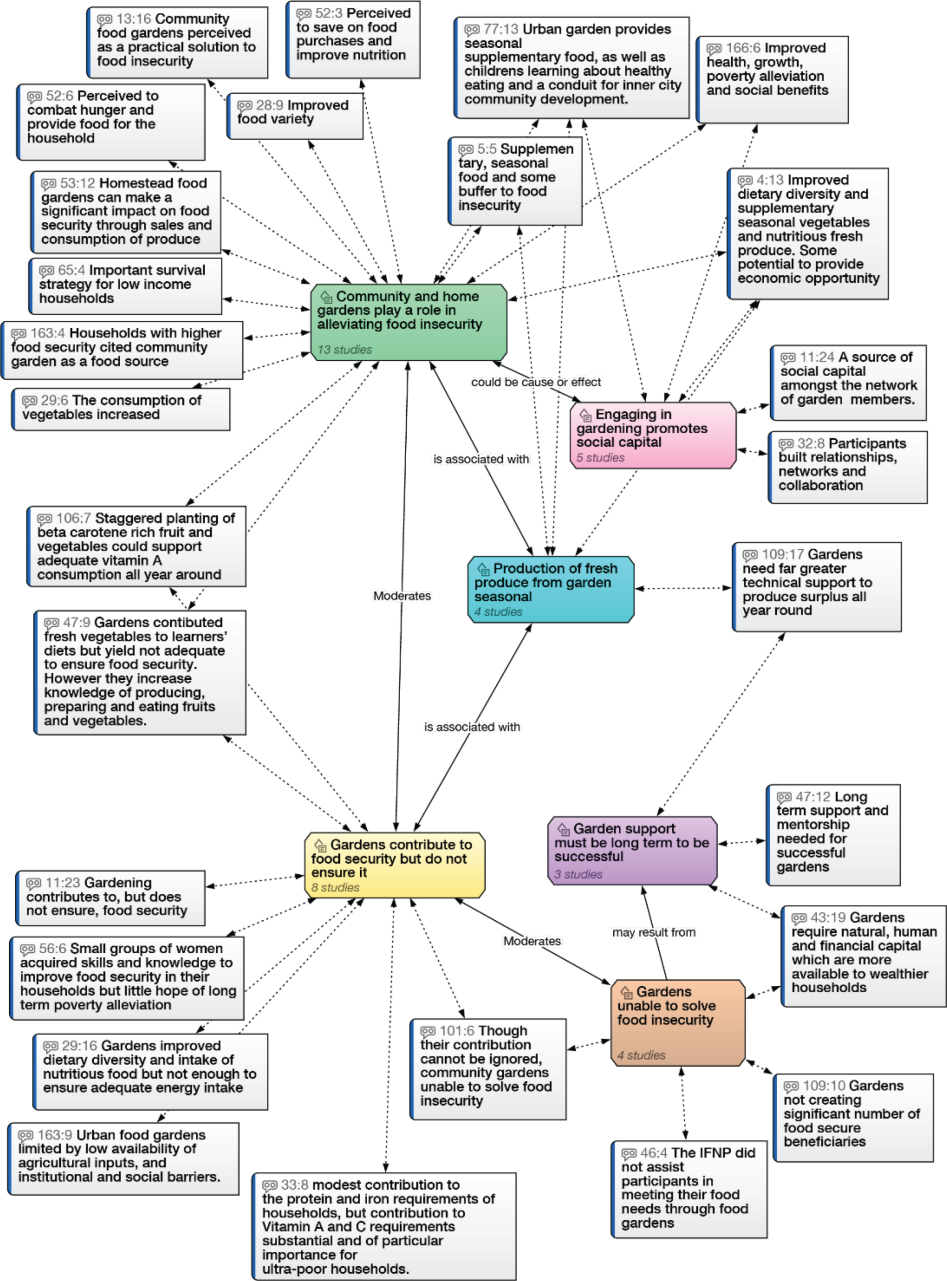
Network diagram showing six themes in the review related to the role of food gardens in food security together with key points noted in the relevant studies.

Of the 169 studies, 23 included a focus on community or homestead gardens, and 13 of these studies were undertaken in urban or peri-urban settings. Six overarching themes emerged in relation to the role of community or homestead gardens in food security, two of which reflect positive impacts resulting from food garden initiatives, and four of which conversely qualify this positive interpretation to varying degrees. A network diagram showing the above themes, together with the paraphrased quotations associated with these themes and their connections, is presented in Fig 7.

*Theme One*: was that gardens played a positive role in alleviating food security (Studies 3, 5, 13, 21, 23, 28, 29, 47, 52, 53, 65, 77, 106, 163, 166) [56, 95–108].

*Theme Two*: highlighted the impact that community gardening had on building various forms of social capital (Studies 3,11, 32, 77, 166) [95, 102, 105, 109, 110].

*Theme Three*: related to the first—was that household and community gardens can contribute to food security, but cannot assure it (Studies 11, 21, 23, 29, 33, 47, 56, 101, 106, 163) [56, 76, 98, 103, 104, 106, 107, 109, 111, 112]

*Theme Four*: Food gardens are frequently only able to provide seasonal fresh produce, which means their role in diet can be erratic (Studies 3, 4, 77, 109) [95, 96, 102, 113].

*Theme Five*: In conflict with more positive findings, however, some studies found that food gardens failed to play any significant role in food security (Studies 43, 46, 101, 109) [71, 76, 84, 113].

*Theme Six*: A final theme was that for gardens to play a decisive role in food security, long term sustained support and/or inputs are required (Studies 21, 43, 47, 109) [84, 98, 106, 113].

The overall picture from the review was that food gardens have the potential to make some contribution to household and community food security, but they require extensive and sustained inputs and/or support to do so. This finding reflects a divergence previously observed in the literature for and against food gardens as a solution to food insecurity (Study 109) [113]. While the role of food gardens in assuring sufficient food may be constrained, the evidence from the review suggests that food gardens can nevertheless play an important part in improving diet quality to include fresh fruit and vegetables–even if only seasonally. They can also contribute to building knowledge about healthy dietary choices, and to building social capital and community development through enhanced networks and cooperation. In the light of the importance of social dynamics noted in the section above on social capital, this is not an insignificant benefit. What remains pivotal is the level of the many resources needed to sustain them.

### What factors moderate the impact of food gardens?

Several studies in the review reveal the impediments to food gardens. In a study examining the impact of the Integrated Food and Nutrition Programme, the limits to food garden contributions to food security were seen to relate to fragmented and inconsistent service provision–such as distribution of agricultural tools without associated training (Study 46) [71]. The difficulty experienced by some gardeners in accessing inputs such as water and suitable land was also noted. The study emphasized the importance of understanding the context and engaging with potential beneficiaries to gather relevant information prior to developing interventions. It was clear that long term sustained support and mentoring increases the likelihood of food garden success in relation to food security; a factor particularly highlighted in a study investigating the impact of school gardens in Johannesburg (Study 47) [98].

The cost related limitations of gardening were frequently noted in the studies, affecting the ability to purchase seed, protect gardens from livestock, and to provide sufficient water. A study examining the role of the retail sector in food security in Mpumalanga noted, “*While a number of rural households still maintain homestead gardens to supplement their diets*, *such gardens are increasingly confined to households at the upper ends of the socioeconomic spectrum with the natural*, *human and financial capital to be able to devote to such non-remunerative productivity*.” (Study 43) [84].

### Urban food systems

Thirty-two of the 169 studies specifically focussed on urban food security, including but not limited to urban food gardens. An overriding message from these studies was that the current urban food system fails to provide food security to the poor in urban areas. This was no surprise in the light of what is known about the high levels of urban food insecurity levels in South Africa [114]. It is estimated (from 2007 data) that more than 50% of the seriously hungry reside in the densely populated urban centres of Cape Town, Ekurhuleni and Johannesburg [65].

The role of urban agriculture, including homestead and community food gardens received mixed treatment–as noted above. A number of prevailing challenges associated with urban agriculture as a solution to food insecurity were reported. As a survivalist strategy amongst the poor, it suffers from the previously highlighted need for both economic and social power to be sustainable—power that the poor seldom have. Review work related to the low-income township of Khayalitsha in Cape Town argues that urban agriculture nevertheless holds potential livelihood benefits, but the need for long-term inputs, particularly under marginal environmental conditions, remains unavoidable [115]. The fragile role of urban agriculture was brought home in a study in Msunduzi in KwaZulu-Natal that drew on data gathered in the 2008–2009 African Food Security Urban Network baseline survey (Study 88) [48]. The survey was administered to a sample of 556 households in a range of lower-income neighborhoods, including new and old townships, informal settlements, peri-urban areas and areas with ‘traditional’ housing. Urban agriculture was found to make only a small contribution to food security, with only 11% of households citing agriculture as a regular food source.

A number of study recommendations look to government to afford this environment, by delivering agricultural assets and land space as well as skills development, educational support, and the removal of institutional barriers (Studies 11, 13, 18, 21, 163) [97, 104, 106, 109, 116]. At the same time, the ability of government to fill this gap apparently remains weak, and without this environment, current urban agriculture is far from being a panacea for the plight of the hungry. The study of Ruysenaar on the role of urban agriculture in food security provides a nuanced critique of the levels of food system understanding and integration, and institutional dynamics required to give urban agriculture a chance of having an effect (Study 109) [113]. He examines the impact of the urban agriculture-orientated programme implemented by the Gauteng Department of Agriculture, Conservation, and Environment. While finding modest benefits, he argues that the safety net functionality of the urban agriculture programme is blurred with income opportunities and that this together with severe operational and institutional shortcomings within the Department confounds any possibility of sustained food security benefits.

### Deagrarianization

Bryceson defined deagrarianization as a process of reorientation of economic activities or livelihoods, changes in occupational activity, and realignment of human settlement away from agrarian patterns. “*Measurable manifestations of this process are*: *a diminishing degree of rural household food and basic needs self-sufficiency*, *a decline in agricultural labor relative to non-agricultural labor in rural households*.” ([117] pg 99]).

One of the arguments put forward for the abandonment of farming following the political transition in the early 1990 is the decrease in government support to former homelands [118]. The cases in the review presented a number of other reasons for the decrease in agricultural production in these areas. One study makes a case for the local availability of high-energy processed foods acting as a disincentive to agricultural production (Study 43) [84]. These authors note: “*an overall trend that grounds many of this study’s findings is the process of deagrarianization together with climate variability and*, *in particular*, *erratic rainfall patterns*” (Study 43) ([84] pg 352]). Other studies find social grants are a disincentive to home farming (Studies 55, 158) [72, 79]. Declining agricultural production is also found in urban and peri-urban agriculture in the Eastern Cape over the ten years prior to the authors’ investigation–i.e. since the mid-1990s (Study 23) [107].

In a study investigating the association between a water irrigation scheme and food security in KwaZulu-Natal, a key point made that the farmer population in the area was aging, with the younger generation looking to move to off-farm ventures which they expect will be more lucrative (Study 45) [119]. This is echoed in studies in the Free State Province (Study 56) [112], KwaZulu-Natal (Studies 90, 168) [93, 120], Limpopo (Study 81) [121], and Eastern Cape (Study 108) [77]. “..*the majority of the rural poor are no longer interested in farming due to poor returns*, *and youths view farming as an unpleasant business enterprise*” (Study 108) ([77] pg, 65). Agricultural activities may even be viewed as stigmatised. For example, a study investigating rainwater harvesting and conservation practices in the Eastern Cape found that food farming is viewed as a signal of poverty, HIV/AIDS, and family abandonment among the community under study (Study 55) [72]: “…*the stigmas of poverty association with food farming also resonate in the wider communities of interests associated with village livelihoods*, *standing and belonging*. *That the majority of food farmers in the two villages are single women and/or aged women*, *enables a further association of food farming with abandonment and isolation*” (Study 55) ([72] pg 11).

### Role of wild food and indigenous crops

A number of studies confirm that wild food sources play a role in the food security of rural households. In the Bushbuckridge District of Limpopo Province, over 91% of households have been found to harvest edible wild plants, 27% of whom consume these daily (Study 113) [122]. Similarly, in northern KwaZulu-Natal, a study across four districts with 99 participants indicated that over 90% consume wild plants, both as a food source and for medicinal purposes (Study 49) [123].

Other studies make a convincing case for wild protein sources being important to nutrition. For example, a study in Eastern Cape Province looking at patterns of food acquisition and consumption among 850 rural children found that 62% of children were supplementing their diets with wild foods, and 30% relied on wild food for more than half their food consumption. Moreover, wild food offered a wider livelihood benefit–particularly for more vulnerable children, with 47% of children selling some of the wild food they collected (Study 135) [124]. Wild food such as insects, birds, and reptiles, was seen as an important protein source for these children, a finding which is in agreement with findings in other studies in the review (Studies 43, 150) [84, 125]. A study in Limpopo Province in two villages found 47 different wild plan species used among 27 families, 95% of which were indigenous. These were used in various roles including firewood, food, and medicine (Study 146) [126].

Wild fruits and leafy vegetables are widely reported on as a long-standing rural food source. A study among 100 rural households in KwaDlangezwa Village in northeast KwaZulu-Natal, an area highly affected by HIV and AIDS, found that the collection of wild spinach was most the commonly used coping strategy in times of hardship and food insecurity (Study 60) [73]. A number of studies in the review confirm that households draw on wild food resources in times of shock, including agricultural related shocks and economic shocks (Studies 3,10, 18, 78, 85) [116, 127–131]. Their availability, however, is markedly seasonal (Studies 73, 153) [63, 132]. In addition to being gathered in the wild, indigenous vegetable crops are also widely consumed by (especially rural) communities in South Africa. These commonly include crops such as sorghum, cowpea, African sweet potatoes, Amaranth leaves, wild pear and buffalo thorn (Study 44) [133].

The level and success of the domestication and consumption of indigenous (sometimes referred to as traditional) plants are often associated with indigenous knowledge (Study 124) [134]. Yet, a number of studies noted that the availability of both wild and cultivated African vegetables have been declining in recent years due not only to declining indigenous knowledge, but to wider cultural changes, and habitat losses (Studies 14, 51, 59, 142, 153, 154) [78, 132, 135–138].

### Gender dynamics

Social resources were frequently linked with leveraging individual power and agency. The position of South African women in the formal and informal institutions in the country has historically left them more vulnerable to food insecurity than men [139]. Food insecurity is embedded in the unequal power women in South Africa have over resources, despite their frequently playing the most active role in providing for household food security. The role of women in agriculture and food security, and their particular powerlessness and vulnerability has been widely researched and reported (e.g. [140]).

**Fig 8:**
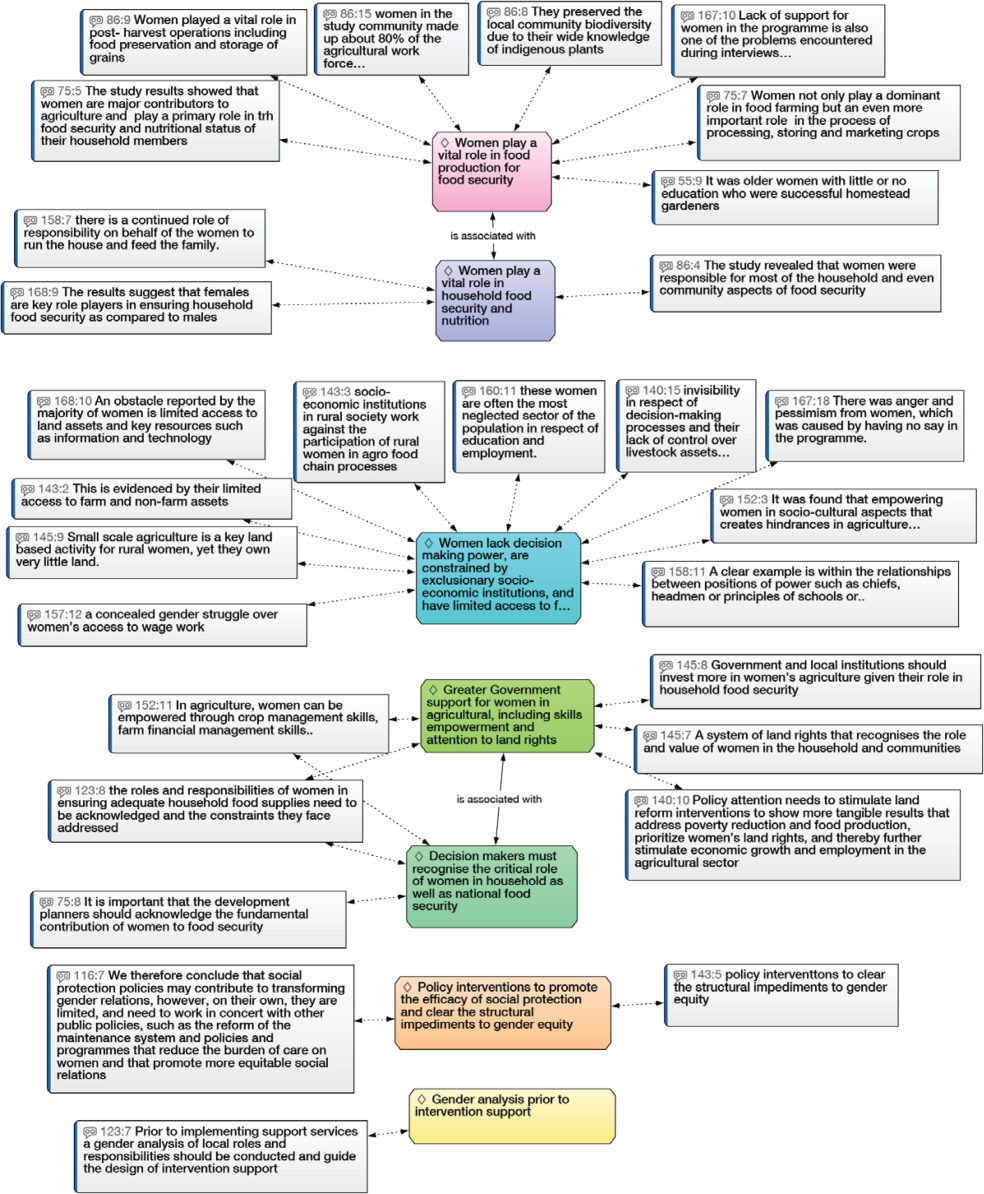
Gender dynamics and food security codes and associated quotations.

The review studies endorse the central role of women in South Africa in the pursuit of household food and security and nutrition (Study 86, 158, 168) [120, 141, 142]. Moreover, household food availability from agricultural production is often driven by women (Studies 55, 75, 86 167) [72, 142–144]. However, the studies find them severely hampered by poor decision-making power, exclusionary socio-economic institutions, and a lack of access to—and control over—both farm and non-farm assets (Studies 19, 140, 143, 145, 152, 157, 158, 160, 168) [82, 120, 141, 145–150]. The review studies do not reveal novel perspectives on the gendered aspects of food insecurity, rather they reinforce and give voice to what is already known. Fig 8 shows the key codes related to gender (in colour), and associated quotations (paraphrased where necessary for brevity) from the studies.

The review studies call for greater government support for women in agriculture and recognition of the fundamental role they play in both household and national food security (Studies 75, 123, 140, 145, 152) [144, 146, 148, 149, 151], as well as attention to the structural hindrances to gender equity (Study 143) [82] such as the important role of social protection, and how this can better work in concert with other government policies (Study 116) [81]. Hart proposes that interventions should also consider existing roles, responsibilities and perspectives to allow for a gendered understanding of food security dynamics prior to being developed (Study 123) [151].

## Short and long term consequences of food insecurity, including coping, adapting and trade-offs

The above sections have explored the body of evidence in the review studies relating to the experience of food insecurity–the most common threads in the tapestry of food insecurity in South Africa. In the following sections, attention is given to two areas that are treated here as key ramifications of these food insecurity threads.

### Coping and not coping

The state of being food insecure is both a cause and consequence of cycles of vulnerability [152]. It is also linked to broader aspects of risk and vulnerability in human-environment interactions (e.g. [153]). Being food insecure is always a result of a resource shortage of one kind or another, which means by definition there are no means for an individual or household to draw on to buffer a food security shock or stressor. Trade-offs result in which people have to either offset short versus long term needs or trade one comfort for another.

The most common response to food shortages found in the review studies was for individuals or households to reduce the frequency, quality or size of their meals. (Studies 1, 2, 3, 10, 13, 15, 16, 18, 19, 41, 43, 59, 66, 76, 82, 96, 101, 108, 160, 169) [49–51, 55, 75–77, 80, 84, 97, 116, 130, 131, 136, 145, 147, 154–157]. This makes intuitive sense in the face of food shortages. However, there are obvious nutritional impacts over time, depending on the extent of deprivation. Other health impacts include the well-documented impact of defaulting on medication if it must be taken with food, as is the case with anti-retroviral which has broad implications in the face of HIV and AIDS pandemic, and/or if there is a cost attached to obtaining it (Studies 20, 160) [145, 158]. Reduced physical function in the face of food shortages (Study 79) [159], increased risk of post-natal depression (Study 117) [160], and fatigue and concentration deficits (Study 35) [161] are also reported across the studies.

There are also other coping strategies frequently employed that have significant implications for deepening vulnerability and reducing resilience in the face of further livelihood stressors. Strategies to procure food such as selling assets (Study 2, 18, 78) [116, 128, 154], for example, or engaging in transaction sex (Study 18, 20, 87) [128, 158, 162], expose individuals to health and livelihood risks and contribute to a downward spiral of vulnerability.

### A nutrition transition

An emerging feature of food insecurity in South Africa are micronutrient deficiencies and overweight malnutrition occurring *alongside* stunting. While dietary preferences play some role, a significant driver of this transition was considered to be the availability of relatively cheap, processed and ‘globalised’ food that is high in energy but generally of poor nutritional value (Studies 15, 30, 38, 62, 79, 98, 141) [53, 55, 58, 60, 159, 163, 164]. Seen in this light, the transition to a globalised diet is not only one of the faces of the experience of food insecurity, but also a consequence of limited resources to support healthy eating.

Overweight, ascribed to the availability of relatively cheap high energy ‘globalised’ food occurred extensively alongside features of food insecurity such as stunting in the studies in this review (studies 15, 30, 38, 62, 79, 98, 141) [53, 55, 58, 60, 159, 163, 164]. The review studies confirm that South African diets are highly dependent on cereals, particularly maize, and frequently lack dietary variety (Studies 2, 3, 8, 43, 62, 68, 84, 99, 109, 144, 149) [47, 62, 84, 113, 131, 154, 163, 165–168].

In one Study among HIV-positive adults included in our review, the prevalence of food insecurity was measured at 70% among the participants—using the Household Food Insecurity Access Scale—yet the prevalence of overweight and obesity were higher than any other form of malnutrition (sample size 300 in a KwaZulu-Natal mixed peri-urban and rural settlement) (Study 62) [163]. Similarly, in a prospective cohort study amongst 162 children in rural Limpopo Province, a high prevalence of stunting (48%) was found (low height for age), together with a high prevalence of overweight (22%) and obesity (24%). Moreover, 19% of the sample were both stunted *and* overweight (Study 98) [53].

A cross-sectional study in 2007, examined a sample of 4000 children in rural Mpumalanga and found 18% stunting among children between the ages of one and four years, with 32% of children at 12 months year being undernourished (Study 30) [60]. Stunting and underweight were also found to be high among adolescent boys, being highest among boys aged 14 years (19%). At the same time, a high prevalence of combined overweight and obesity was found among adolescent girls, reaching a peak at aged 18 years (25%).

A study among children attending primary schools in the Western Cape found that 2% of children were underweight, 19% were stunted, and 20% were overweight or obese. Learners with a lower standard of living scores were those that were more likely to purchase unhealthy food items from a food vendor for lunch rather than carry a lunchbox to school. These learners were also more likely to be overweight or obese (Study 141) [58].

A number of studies included in the review reported the observation that ‘high energy foods’ were eaten in preference to high-nutrient content foods (studies 15, 38, 43, 47) [55, 84, 98, 164]. In addition to the issue of overweight, the issue of micronutrient malnutrition is also raised in the review studies. One study that included a sample of 285 institutionalised and community-dwelling black South African men and women over the age of 60 found no association between BMI and added sugar intake, but reported that: "Our data have demonstrated that in elderly South African women, but not men, who have little financial means, the nutrient-diluting effect of a high sugar consumption places them at high risk for inadequate micronutrient, protein and fibre intakes" (Study 79) ([159] pg 104).

Echoing this, in a cross-sectional study of 136 adults, in rural KwaZulu-Natal, the authors concluded that there was excess caloric intake *together with* inadequate micronutrient inadequacies in rural South African communities (Study 93) [169]. This study made use of a 24-hour recall tool, together with food quantities, and also collected 16 composite dishes from the community for nutritional analysis.

Food availability in local markets does not necessarily translate into food security for those accessing it. Available alternative ‘globalised’ foods offer cheaper or more appealing options to consumers. A thesis study included in the review lends support to this notion. The study looked at the impact of the government’s Nutritional Supplementation Programme on children’s food security using a mixed methodology, reporting “… *no association between food access (FA) and nutritional security (NS)*, *meaning that food security does not automatically translate to nutritional security*” (Study 15) ([55] pg. i). This is a bold statement, but one that may need to be taken quite seriously in the consideration of food security in South Africa. The definition of food security does, in fact, *include* nutrition, but the authors nevertheless emphasize the transition in food insecurity focus being from accessing ‘enough food’ to accessing ‘enough of the right kind of food' for many of the poor in South Africa.

A cross-sectional, comparative, population-based study was undertaken in North West Province among 1854 participants from 37 different locations [170]. The survey looked at a range of socio-economic indicators as well as nutrition. The risk of non-communicable disease was assessed through measurement of 50 discrete biological variables indicative of—for example–diseases such as diabetes mellitus. The findings suggest that a ‘nutrition transition' does not necessarily lead to a reduced intake of micronutrients. Rather, the authors find that urbanization improved micronutrient intake, but also increased the risk of overweight and obesity, and–perhaps even more importantly–the risk of non-communicable disease [170].

## Implications for policy and practice

The recommendations from the studies included in this review were surprisingly scattered and generally weak. About 47% of the studies do not make any specific recommendations for policy or programming based on their work. The remainder largely makes broad comments that have limited currency in terms of translation into specific recommendations.

An area of particular emphasis was in recommendations related to agriculture and food gardens. No less than 40 papers of the 169 (about 24%) in the systematic review provide recommendations linked to improving various forms of support for agriculture or food gardening as a means to address food insecurity. Of these papers, 38 were specifically investigating agriculture or food gardening. It is clear that agriculture remains a high-level focus in regard to food security in South Africa, and that it is an area with many challenges.

There was a strong focus on better governmental department or institutional coordination (Studies 46, 72, 92, 109, 118, 122, 123, 140) [71, 113, 121, 146, 151, 171–173], which is a recommendation already given much treatment in food security literature in South Africa. Six of eight studies specifically explored government programming or policies.

Among the studies that expressly explored gender issues, there was a concomitant call for acknowledgment of, and support for, the role of women in agriculture, including their land rights (Studies 75, 123, 140, 145, 152) [146, 148, 149, 151, 174].

In urban areas, the provision of urban agricultural land space and agricultural assets was strongly emphasized (studies 11, 13, 21) [97, 106, 109]. Notwithstanding the mixed evidence for their efficacy, in both rural and urban areas state support for community or household food gardens was a focus in a number of studies (studies 1, 4, 5, 13, 15, 18, 47, 76) [49, 51, 55, 95–98, 116]. Improved agricultural extension services were seen as critical for agricultural endeavors generally (studies 45, 123, 129, 162) [119, 151, 175, 176].

A number of studies stressed the importance of support for low-technology approaches to agriculture which are appropriate to the context and environmental conditions. These included low-input agriculture (Studies 125, 142, 159) [78, 177, 178] as well as farming indigenous crop varieties for their local-level environmental adaptability. The nutritional value was also highlighted (Studies 44, 51, 86, 97, 124) [133–135, 179, 180]. However, the above recommendations need to be seen in the light of declining engagement in small-scale agriculture as outlined in the section on deagrarianisation above.

## Conclusions

The experience of food insecurity found in this review was characterised by issues affecting the ability of individuals and households to access sufficient food. This resonated with–and expands on—the shift in food security ‘paradigms’ narrated on in the 1990s by Maxwell, who observed that since the 1970s the focus in analysis and programming has moved from being on national production deficits to being orientated towards the consumer, at the level of household and individual livelihoods and capabilities (see [181]). These shifts have also seen the commensurate change in the way food security is measured, and how best to do so has been the subject of scholarly debate for some time [37, 182].

The use of 27 different measurement tools across the 169 studies is indicative not only of the different disciplinary backgrounds of the authors but also of the difficulty in establishing an absolute ‘state' of food insecurity that is comparable across space and time. Some approaches focussed on measuring food security through a composite food access index, others on quantifying micro- and macro-nutrient intake, and still others on malnutrition outcomes such as stunting. This makes a direct comparison of relative food insecurity across the studies very difficult.

A wide range of food insecurity drivers was cited across the studies, with each study reflecting local level, context-specific dynamics. Nevertheless, the very high reliance on purchased food means that being able to afford food is the predominant challenge in South Africa, with 51% of studies specifically citing poverty as a food insecurity driver. Food prices are a cause for concern and discussion. In the light of poverty, there is a high dependence on government grants, which is seen as preventing households from falling into absolute hunger, but may be a disincentive to engaging in more sustainable food security efforts, such as food gardens. This is resonant with the kinds of trade-offs involved among the food insecure, in which coping strategies frequently have short as well as long-term negative impacts on livelihood resilience.

Food production is beset with access-related issues including affordability of agricultural inputs, poor access to farm and non-farm resources, and failing or exclusionary formal and informal institutions. Given these challenges, it is perhaps not surprising that deagrarianisation emerged as a theme in the studies. Deagrarianisation also takes place in the context of a rapidly urbanising world. Urban food systems were discussed by some studies.

The exclusionary practices of formal and informal institutions were repeatedly raised in relation to agriculture and other aspects of food access. Access to social resources was particularly mentioned in relation to gender dynamics, which disadvantage women in myriad ways in the food security landscape.

The so-called nutrition transition creates a (relatively new) dimension to food insecurity, and one that is linked to markets and food availability, food choices and nutrition, as well as food access. This transition sees a move towards the increasing consumption of cheaper, more available, and/or preferable ‘globalised’ foods, which have a high-energy content but generally low nutritional value, and which are heralding overweight and obesity alongside features of malnutrition such as stunting. However, all of the food security issues raised in the studies, are components of a food system that has multiple ailments but also multiple opportunities.

A high level of local specificity in the causes, experience and consequences of food insecurity in South Africa is evident from the reviewed research. Decision-makers need to consider the local level context in developing and implementing interventions. From an operational perspective, the ‘how’ of interventions- their process- matters at least as much as their ‘what’. Misselhorn’s framework for intervention processes incorporates *social capital*, *participation*, *coordination*, and *learning interactions* as essential elements in food security interventions [183].

The study highlights the need for paying more attention to what we are measuring and being precise in how food insecurity is measured. While there are no agreed on measures internationally, it is important for the food security research community and national departments entrusted with monitoring and evaluation of food security (primarily the Departments for Planning, Monitoring and Evaluation; Agriculture, Forestry and Fisheries; Health; Rural Development and Land Reform and Statistics South Africa) to confer and agree on a minimum set of indicators, methodologies and the interpretation of these indicators for reporting in more consistent ways. The wealth of sub-national surveys could provide additional rich understandings of local and household level experiences of food security and informed evidence for more appropriate policy making and designing of intervention programmes. Policymakers at all spheres of government (national, provincial and municipal) should be engaging with this wealth of researchers and sub-national study findings to enrich their food security planning, monitoring, and evaluation.

## Supporting information

S1 Appendix List of case studies. (DOCX)

S1 File Annex of terms. (DOCX)

S1 Checklist PRISMA 2009 checklist. (DOC)
