# Supplementary material for: A systematic review of sub-national food insecurity research in South Africa: Missed opportunities for policy insights
Source: PLoS One. 2017 Aug 22;12(8):e0182399. doi: 10.1371/journal.pone.0182399 (PMC5567909; doi:10.1371/journal.pone.0182399)
Supplement: S1 Appendix — (DOCX) [file pone.0182399.s001.docx]

**S1 Atlas.ti Report with List of Case Studies and Project Reference Numbers**

Note: Each document appears with an Atlas.ti reference number. This number corresponds to the numbers in the top left corner of the quotations that appear in the Atlas.ti network diagrams in this report.

A = Aims

L = Location

M = Food security measure used

P = Province

R = Rating for relevance and validity for purposes of THIS review

S = Sample size

T = Type of document

**
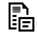
 1 Walsh_2014 et al.pdf**

**Groups:**


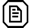
 2014 
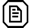
 A-State of food insecurity and/or nutrition 
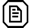
 L-Participants drawn from urban and rural 
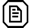
 M-HFIAS 
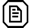
 P-Free State 
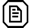
 R- 13-15 Good 
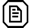
 S->500 
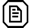
 T-Peer reviewed journal article

**
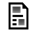
 2 Lushaba 2005 thesis.pdf**

**Groups:**


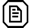
 2005 
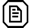
 A-Impact of HIV and AIDS and/or links with food security 
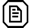
 L-Rural 
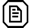
 P-KwaZulu Natal 
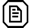
 R- 9-12 Moderate 
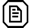
 S-0-50 
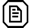
 T-Thesis or research report

**
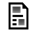
 3 Majake 2011 thesis.pdf**

**Groups:**


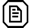
 2005 
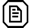
 A-Impact Assessment or Feasibility Study 
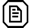
 L-Rural 
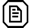
 M-Coping strategy index 
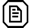
 M-Food frequency questions 
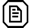
 P-Free State 
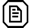
 R- 13-15 Good 
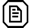
 S-51-100 
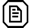
 T-Thesis or research report

**
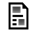
 4 Mudzinganyama 2012 thesis.pdf**

**Groups:**


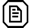
 2012 
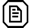
 A-Contribution of food gardens to food security 
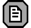
 A-Role of community or homestead gardens in food security 
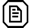
 L-Rural 
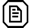
 M-Food Expenditure 
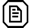
 M-Seasonal calendar 
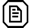
 P-KwaZulu Natal 
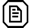
 R- 13-15 Good 
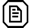
 S-0-50 
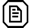
 T-Thesis or research report

**
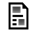
 5 Ngidi 2007 thesis.pdf**

**Groups:**


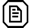
 2007 
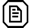
 A-Contribution of production to food security 
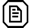
 L-Rural 
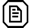
 M-Coping strategy index 
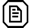
 P-KwaZulu Natal 
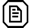
 R- 13-15 Good 
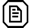
 S-201-500 
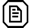
 T-Thesis or research report

**
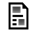
 6 Mosisi 2009 thesis.pdf**

**Groups:**


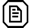
 2009 
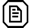
 A-Impact Assessment or Feasibility Study 
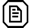
 L-Peri-urban 
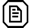
 M-Household dietary diversity 7 day recall 
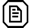
 P-KwaZulu Natal 
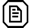
 R- 9-12 Moderate 
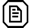
 S-0-50 
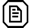
 T-Thesis or research report

**
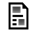
 7 Cresswilliams 2001 thesis.pdf**

**Groups:**


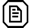
 2001 
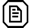
 A-Impact Assessment or Feasibility Study 
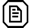
 L-Urban 
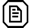
 P-Eastern Cape 
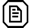
 R - 0-8 Low 
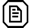
 S-101-200 
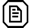
 T-Thesis or research report

**
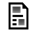
 8 Khanyile 2012 thesis.pdf**

**Groups:**


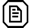
 2012 
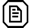
 A-Contribution of food gardens to food security 
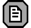
 A-Role of community or homestead gardens in food security 
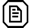
 L-Rural 
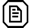
 P-KwaZulu Natal 
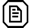
 R- 9-12 Moderate 
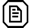
 S-0-50 
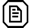
 T-Thesis or research report

**
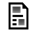
 9 Floersch 2012 thesis.pdf**

**Groups:**


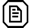
 2011 
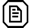
 A-Youth's perspectives on food security 
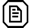
 L-Rural 
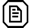
 P-KwaZulu Natal 
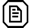
 R- 13-15 Good 
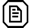
 S-0-50 
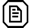
 T-Thesis or research report

**
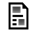
 10 Schroeder 2003 thesis.pdf**

**Groups:**


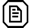
 2003 
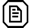
 A-Impact of HIV and AIDS and/or links with food security 
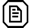
 L-Rural 
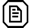
 M-Food grown or production deficit 
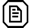
 P-KwaZulu Natal 
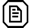
 R- 13-15 Good 
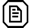
 S-51-100 
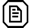
 T-Thesis or research report

**
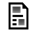
 11 Mthethwa 2012 thesis.pdf**

**Groups:**


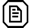
 2012 
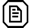
 A-Role of urban agriculture in livelihoods and food security 
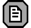
 A-Urban food system 
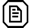
 L-Urban 
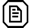
 M-Food Expenditure 
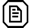
 M-Self assessed food security 
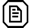
 P-KwaZulu Natal 
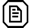
 R - 16-18 Excellent 
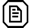
 S-0-50  T-Thesis or research report

**12 Roos 2012 thesis.pdf**

**Groups:**

2012  A-Local urban food system  A-Urban food system  L-Urban  P-Western Cape  R- 13-15 Good  S-201-500  T-Thesis or research report

**13 Madlala 2012 thesis.pdf**

**Groups:**

2012  A-Contribution of food gardens to food security  A-Perceptions and experience of food security  A-Role of community or homestead gardens in food security  L-Peri-urban  P-KwaZulu Natal  R- 13-15 Good  S-0-50  T-Thesis or research report

**14 Vorster et al 2008.pdf**

**Groups:**

2008  A-Role of traditional green leafy vegetables in food security  A-Wild foods, traditional indigenous food, indigenous knowledge  L-Rural  P-KwaZulu Natal  P-Limpopo  R- 9-12 Moderate  S-101-200  T-Peer reviewed journal article

**15 Tshabalala 2014.pdf**

**Groups:**

2014  A-Impact Assessment or Feasibility Study  L-Participants drawn from urban and rural  M-Anthropometric measures  M-Child food insecurity access scale  M-Food Expenditure  M-HFIAS  M-Household Dietary Diversity 24 hour recall  M-Months adequate food provisioning  P-KwaZulu Natal  R - 16-18 Excellent  S-101-200  T-Thesis or research report

**16 Msaki 2010 thesis.pdf**

**Groups:**

2010  A-Development of food security measurement tool/s  L-Rural  M-Coping strategy index  M-Food Expenditure  M-Household Dietary Diversity 24 hour recall  P-KwaZulu Natal  R - 16-18 Excellent  S-101-200  T-Thesis or research report

**17 Abdulla-Merzouk 2008 thesis.pdf**

**Groups:**

2008  A-Contribution of street trading to food security of traders  L-Urban  M-Coping strategy index  P-KwaZulu Natal  R- 9-12 Moderate  S-0-50  T-Thesis or research report

**18 Mjonono 2008 thesis.pdf**

**Groups:**

2008  A-Coping strategies  A-Shocks, stressors, coping strategies  L-Rural  M-Coping strategy index  P-KwaZulu Natal  R- 9-12 Moderate  S-101-200  T-Thesis or research report

**19 Dlamini and Tabit 2014.pdf**

**Groups:**

2014  A-Coping strategies  A-Shocks, stressors, coping strategies  L-Rural  M-Coping strategy index  P-Eastern Cape  R - 0-8 Low  S-51-100  T-Peer reviewed journal article

**20 Nyakurimwa 2011 thesis.pdf**

**Groups:**

2011  A-Perceptions and experience of food security  L-Rural  P-KwaZulu Natal  R- 9-12 Moderate  S-0-50  T-Thesis or research report

**21 Pillay 2002 thesis.pdf**

**Groups:**

2002  A-Role of urban agriculture in livelihoods and food security  A-Urban food system  L-Urban  P-KwaZulu Natal  R- 9-12 Moderate  S-0-50  T-Thesis or research report

**23 Thornton and Nel 2007.pdf**

**Groups:**

2007  A-Role of urban agriculture in livelihoods and food security  A-Urban food system  L-Peri-urban  P-Eastern Cape  R - 0-8 Low  S-0-50  T-Peer reviewed journal article

**24 Ngidi 1997 thesis.pdf**

**Groups:**

1997  A-Contribution of production to food security  L-Rural  P-KwaZulu Natal  R- 9-12 Moderate  S-0-50  T-Thesis or research report

**25 phometsi 2004 thesis.pdf**

**Groups:**

2004  A-Impact Assessment or Feasibility Study  L-Rural  M-Nutrition knowledge  P-North West  R- 13-15 Good  S-101-200  T-Thesis or research report

**26 Maferetlhane 2012 thesis.pdf**

**Groups:**

2012  A-Role of indigenous knowledge  A-Wild foods, traditional indigenous food, indigenous knowledge  L-Rural  P-North West  R- 9-12 Moderate  S-0-50  T-Thesis or research report

**27 Ghebremicael 2000 thesis.pdf**

**Groups:**

2000  A-Role of urban agriculture in livelihoods and food security  A-Urban food system  L-Peri-urban  P-KwaZulu Natal  R- 13-15 Good  S-0-50  T-Thesis or research report

**28 Ndlovu 2007 thesis.pdf**

**Groups:**

2007  A-Community gardens and homestead gardens  A-Role of community or homestead gardens in food security  L-Rural  P-KwaZulu Natal  R- 9-12 Moderate  S-0-50  T-Thesis or research report

**29 Selepe 2010 thesis.pdf**

**Groups:**

2010  A-Contribution of food gardens to food security  A-Impact Assessment or Feasibility Study  A-Role of community or homestead gardens in food security  L-Peri-urban  M-Anthropometric measures  M-Food frequency questions  M-Household Dietary Diversity 24 hour recall  P-Gauteng  R- 13-15 Good  S-0-50  T-Thesis or research report

**30 Kimani 2010 thesis.pdf**

**Groups:**

2010  A-Explore malnutrition and or its impacts  A-Impact of HIV and AIDS and/or links with food security  L-Rural  M-Anthropometric measures  P-Mpumalanga  R - 16-18 Excellent  S->500  T-Thesis or research report

**31 Owen and Goldin 2015.pdf**

**Groups:**

2013  A-Exploring capabilities and food security  L-Rural  M-Months adequate food provisioning  P-Mpumalanga  R- 9-12 Moderate  S-201-500  T-Peer reviewed journal article

**32 van averbeke 2007.pdf**

**Groups:**

2007  A-Role of urban agriculture in livelihoods and food security  A-Urban food system  L-Urban  P-Gauteng  R- 9-12 Moderate  S-101-200  T-Peer reviewed journal article

**33 van Averbeke and Khosa 2007.pdf**

**Groups:**

2007  A-Contribution of food gardens to food security  A-Role of community or homestead gardens in food security  L-Rural  M-Food Expenditure  M-Food grown or production deficit  P-Limpopo  R- 9-12 Moderate  S-101-200  T-Peer reviewed journal article

**34 Ndwandwe and Mudhara 2014.pdf**

**Groups:**

2014  A-Role of indigenous knowledge  A-Wild foods, traditional indigenous food, indigenous knowledge  L-Rural  M-Availability of produced maize  P-KwaZulu Natal  R - 0-8 Low  S-51-100  T-Peer reviewed journal article

**35 Munro et al 2013.pdf**

**Groups:**

2013  A-State of food insecurity and/or nutrition  L-Urban  M-Students food insecurity questionnaire  P-KwaZulu Natal  R- 13-15 Good  S->500  T-Peer reviewed journal article

**36 Balogun et al 2015.pdf**

**Groups:**

2014  A-State of food insecurity and/or nutrition  L-Urban  M-Food frequency questions  M-Food quantiy estimates and nutrient content  P-Western Cape  R- 13-15 Good  S-201-500  T-Peer reviewed journal article

**37 Tshitangano et al 2014.pdf**

**Groups:**

2014  A-Nutrition knowledge  L-Rural  P-Limpopo  R- 9-12 Moderate  S-201-500  T-Peer reviewed journal article

**38 Audain et al 2014.pdf**

**Groups:**

2014  A-Food preferences  L-Participants drawn from urban and rural  M-Food frequency questions  P-KwaZulu Natal  R- 13-15 Good  S-101-200  T-Peer reviewed journal article

**39 Smit_2015.pdf**

**Groups:**

2014  A-Local urban food system  A-Urban food system  L-Urban  P-Western Cape  R- 9-12 Moderate  S-0-50  T-Peer reviewed journal article

**40 May_2014.pdf**

**Groups:**

2014  A-State of food insecurity and/or nutrition  L-Participants drawn from urban and rural  M-Food quantiy estimates and nutrient content  M-Household Dietary Diversity 24 hour recall  P-Western Cape  R - 16-18 Excellent  S-51-100  T-Peer reviewed journal article

**41 Grobler 2014.pdf**

**Groups:**

2014  A-Coping strategies  A-Shocks, stressors, coping strategies  L-Urban  M-Coping strategy index  M-HFIAS  P-Gauteng  R- 9-12 Moderate  S-201-500  T-Peer reviewed journal article

**42 Kepe_2014.pdf**

**Groups:**

2014  A-Land rights  L-Rural  P-Eastern Cape  R - 0-8 Low  S-0-50  T-Thesis or research report

**43 Pereira et al 2014.pdf**

**Groups:**

2014  A-Contribution of production to food security  A-Coping strategies  A-Role of private sector and retail in food security  A-Shocks, stressors, coping strategies  L-Rural  M-Household Dietary Diversity 24 hour recall  P-Mpumalanga  R - 16-18 Excellent  S-101-200  T-Peer reviewed journal article

**44 Cloete and Idsardi 2013.pdf**

**Groups:**

2013  A-Consumption / role of traditional and indigenous crops  A-Wild foods, traditional indigenous food, indigenous knowledge  L-Participants drawn from urban and rural  P-North West  R- 9-12 Moderate  S->500  T-Peer reviewed journal article

**45 Sinyolo 2014.pdf**

**Groups:**

2014  A-Agriculture, agribusiness and agricultural extension  A-Water security and food security  L-Rural  M-Food Expenditure  P-KwaZulu Natal  R - 16-18 Excellent  S-101-200  T-Peer reviewed journal article

**46 Prinsloo and pillay 2014.pdf**

**Groups:**

2014  A-Impact Assessment or Feasibility Study  L-Peri-urban  P-Gauteng  R- 9-12 Moderate  S-51-100  T-Peer reviewed journal article

**47 Beery et al 2013.pdf**

**Groups:**

2013  A-Contribution of food gardens to food security  A-Impact Assessment or Feasibility Study  A-Role of community or homestead gardens in food security  L-Urban  M-Food quantiy estimates and nutrient content  M-Household Dietary Diversity 24 hour recall  P-Gauteng  R- 9-12 Moderate  S-51-100  T-Peer reviewed journal article

**48 Dzerefos et al 2014.pdf**

**Groups:**

2014  A-Wild foods  A-Wild foods, traditional indigenous food, indigenous knowledge  L-Rural  P-Limpopo  R - 0-8 Low  S-101-200  T-Peer reviewed journal article

**49 Lewu and Mavengahama 2011.pdf**

**Groups:**

2011  A-Wild foods  A-Wild foods, traditional indigenous food, indigenous knowledge  L-Rural  P-KwaZulu Natal  R- 9-12 Moderate  S-51-100  T-Peer reviewed journal article

**50 Ndobo and Sekhampu 2013.pdf**

**Groups:**

2013  A-Causes of food insecurity  L-Peri-urban  M-HFIAS  P-Free State  R- 13-15 Good  S-201-500  T-Peer reviewed journal article

**51 Dweba and mearns 2011.pdf**

**Groups:**

2011  A-Role of indigenous knowledge  A-Wild foods, traditional indigenous food, indigenous knowledge  L-Rural  P-Eastern Cape  R- 9-12 Moderate  S-0-50  T-Peer reviewed journal article

**52 Tembo_2013.pdf**

**Groups:**

2013  A-Community gardens and homestead gardens  A-Contribution of food gardens to food security  A-Role of community or homestead gardens in food security  L-Urban  P-Western Cape  R- 9-12 Moderate  S-0-50  T-Peer reviewed journal article

**53 Botha_2012.pdf**

**Groups:**

2012  A-Community gardens and homestead gardens  A-Impact Assessment or Feasibility Study  A-Role of community or homestead gardens in food security  L-Rural  M-Food Expenditure  M-Food grown or production deficit  P-Free State  R- 9-12 Moderate  S-51-100  T-Peer reviewed journal article

**54 Tshuma and Monde 2012.pdf**

**Groups:**

2012  A-Impact Assessment or Feasibility Study  L-Rural  M-Self assessed food security  P-Eastern Cape  R- 9-12 Moderate  S-0-50  T-Peer reviewed journal article

**55 Minkley_2012.pdf**

**Groups:**

2012  A-Agriculture, agribusiness and agricultural extension  A-Community gardens and homestead gardens  A-Impact Assessment or Feasibility Study  A-Role of community or homestead gardens in food security  A-Water security and food security  L-Rural  M-Not directly measured or measure unspecified  P-Eastern Cape  R - 16-18 Excellent  S-51-100  T-Peer reviewed journal article

**56 Esterhuyse_2012.pdf**

**Groups:**

2012  A-Agriculture, agribusiness and agricultural extension  A-Community gardens and homestead gardens  A-Impact Assessment or Feasibility Study  A-Role of community or homestead gardens in food security  A-Water security and food security  L-Rural  P-Free State  R- 13-15 Good  S-not specified  T-Peer reviewed journal article

**57 DeSilva et al 2015.pdf**

**Groups:**

2012  A-Impact of HIV and AIDS and/or links with food security  L-Participants drawn from urban and rural  M-Anthropometric measures  M-Food frequency questions  M-Self assessed food security  P-KwaZulu Natal  R - 16-18 Excellent  S->500  T-Peer reviewed journal article

**58 Kaschula and Shackleton 2012.pdf**

**Groups:**

2012  A-Impact of HIV and AIDS and/or links with food security  A-Wild foods  A-Wild foods, traditional indigenous food, indigenous knowledge  L-Rural  P-Eastern Cape  P-KwaZulu Natal  R- 13-15 Good  S->500  T-Peer reviewed journal article

**59 Quinn et al 2011.pdf**

**Groups:**

2011  A-Coping strategies  A-Shocks, stressors, coping strategies  L-Rural  P-Limpopo  R- 9-12 Moderate  S-51-100  T-Peer reviewed journal article

**60 Kaschula 2011.pdf**

**Groups:**

2011  A-Coping strategies  A-Impact of HIV and AIDS and/or links with food security  A-Shocks, stressors, coping strategies  L-Rural  M-Coping strategy index  M-Household dietary diversity 48hour recall  P-KwaZulu Natal  R - 16-18 Excellent  S-51-100  T-Peer reviewed journal article

**61 Goudge_2011.pdf**

**Groups:**

2011  A-Impact of HIV and AIDS and/or links with food security  L-Urban  P-Gauteng  R - 0-8 Low  S-0-50  T-Peer reviewed journal article

**62 Oketch_2011.pdf**

**Groups:**

2011  A-Impact Assessment or Feasibility Study  A-Impact of HIV and AIDS and/or links with food security  L-Participants drawn from urban and rural  M-Anthropometric measures  M-HFIAS  M-Household Dietary Diversity 24 hour recall  M-Nutrition knowledge  M-Self assessed food security  P-KwaZulu Natal  R- 13-15 Good  S-201-500  T-Peer reviewed journal article

**63 Andresen et al 2009.pdf**

**Groups:**

2009  A-Impact Assessment or Feasibility Study  L-Urban  P-Western Cape  R- 9-12 Moderate  S-0-50  T-Peer reviewed journal article

**64 Venter et al 2009.pdf**

**Groups:**

2009  A-Impact of HIV and AIDS and/or links with food security  L-Urban  M-Anthropometric measures  M-Healthy eating index  P-Gauteng  R- 9-12 Moderate  S-51-100  T-Peer reviewed journal article

**65 Thornton_2009.pdf**

**Groups:**

2009  A-Land rights  L-Peri-urban  P-Eastern Cape  R - 0-8 Low  S-not specified  T-Peer reviewed journal article

**66 Akinboade 2008.pdf**

**Groups:**

2008  A-Impact of HIV and AIDS and/or links with food security  A-Land rights  L-Rural  P-Limpopo  R- 13-15 Good  S-51-100  T-Peer reviewed journal article

**67 Faber et al 2007.pdf**

**Groups:**

2007  A-Role of traditional green leafy vegetables in food security  A-Wild foods, traditional indigenous food, indigenous knowledge  L-Rural  M-Food quantiy estimates and nutrient content  M-Household Dietary Diversity 24 hour recall  P-KwaZulu Natal  R- 13-15 Good  S-51-100  T-Peer reviewed journal article

**68 Hattingh_2006.pdf**

**Groups:**

2006  A-Impact of HIV and AIDS and/or links with food security  L-Urban  M-Food frequency questions  M-Food quantiy estimates and nutrient content  P-Free State  R- 13-15 Good  S-201-500  T-Peer reviewed journal article

**69 Lemke et al 2003.pdf**

**Groups:**

2003  A-Causes of food insecurity  L-Participants drawn from urban and rural  M-Food Expenditure  P-North West  R- 13-15 Good  S-101-200  T-Peer reviewed journal article

**70 Le-Roy_2000.pdf**

**Groups:**

2000  A-Development of food security measurement tool/s  L-Rural  M-Food Expenditure  M-Food grown or production deficit  M-Food quantiy estimates and nutrient content  P-Limpopo  R- 9-12 Moderate  S-51-100  T-Peer reviewed journal article

**71 McCuster 1997 thesis.pdf**

**Groups:**

1997  A-Causes of food insecurity  A-Coping strategies  A-Shocks, stressors, coping strategies  A-State of food insecurity and/or nutrition  L-Rural  M-Months adequate food provisioning  P-KwaZulu Natal  R- 9-12 Moderate  S-51-100  T-Thesis or research report

**72 Matjokana_2013.pdf**

**Groups:**

2013  A-Impact Assessment or Feasibility Study  L-Rural  P-Limpopo  R - 0-8 Low  S-not specified  T-Thesis or research report

**73 Matla 2008 thesis.pdf**

**Groups:**

2008  A-Impact Assessment or Feasibility Study  L-Rural  M-Anthropometric measures  P-Free State  R- 13-15 Good  S-0-50  T-Thesis or research report

**74 Mbele 2009 thesis.pdf**

**Groups:**

2009  A-Agriculture, agribusiness and agricultural extension  A-Biofuels and food security  L-Rural  P-KwaZulu Natal  R - 0-8 Low  S-0-50  T-Thesis or research report

**75 Raidimi 2014.pdf**

**Groups:**

2014  A-Role of women in food security  A-Women or gender and food security  L-Rural  P-Limpopo  R- 9-12 Moderate  S-201-500  T-Peer reviewed journal article

**76 Ballantine 2007 thesis.pdf**

**Groups:**

2007  A-Causes of food insecurity  L-Rural  M-HFIAS  P-Eastern Cape  R- 13-15 Good  S-201-500  T-Thesis or research report

**77 Trixie Belle 2011.pdf**

**Groups:**

2011  A-Role of urban agriculture in livelihoods and food security  A-Urban food system  L-Urban  P-Gauteng  R- 13-15 Good  S-0-50  T-Peer reviewed journal article

**78 Akpalu 2005 thesis.pdf**

**Groups:**

2005  A-Coping strategies  A-Shocks, stressors, coping strategies  L-Rural  P-Limpopo  R- 13-15 Good  S-0-50  T-Thesis or research report

**79 Charlton et al 2005.pdf**

**Groups:**

2005  A-State of food insecurity and/or nutrition  L-Urban  M-Blood tests  M-Household Dietary Diversity 24 hour recall  M-Mini nutritional assessment  P-Not specified  R- 13-15 Good  S-201-500  T-Peer reviewed journal article

**80 Dodd and Nyabvudzi 2014.pdf**

**Groups:**

2014  A-Causes of food insecurity  L-Rural  M-Household hunger scale  P-Eastern Cape  R - 0-8 Low  S-201-500  T-Peer reviewed journal article

**81 Drimie et al 2013.pdf**

**Groups:**

2013  A-Dietary diversity and food security  A-State of food insecurity and/or nutrition  L-Urban  M-Household Dietary Diversity 24 hour recall  P-Limpopo  R- 13-15 Good  S-101-200  T-Peer reviewed journal article

**82 Masekoameng and Maliwichi 2014.pdf**

**Groups:**

2014  A-Causes of food insecurity  L-Rural  M-HFIAS  P-Limpopo  R - 0-8 Low  S->500  T-Peer reviewed journal article

**83 Oldewage-Theron and Egal 2012.pdf**

**Groups:**

2012  A-Impact Assessment or Feasibility Study  L-Rural  P-Free State  R- 9-12 Moderate  S-51-100  T-Peer reviewed journal article

**84 Sheehy_et_al-2014-.pdf**

**Groups:**

2014  A-Development of food security measurement tool/s  L-Rural  M-Household Dietary Diversity 24 hour recall  P-KwaZulu Natal  R- 9-12 Moderate  S-51-100  T-Peer reviewed journal article

**85 Tibesigwa et al 2015.pdf**

**Groups:**

2014  A-Agricultural shocks and food security  A-Coping strategies  A-Shocks, stressors, coping strategies  L-Rural  M-Monthly per capita calorie consumption  P-Mpumalanga  R- 13-15 Good  S->500  T-Peer reviewed journal article

**86 Tlhompho 2014.pdf**

**Groups:**

2014  A-Explore climate change or variability and food security  A-Perceptions and experience of food security  A-Role of indigenous knowledge  A-Shocks, stressors, coping strategies  A-Wild foods, traditional indigenous food, indigenous knowledge  L-Rural  P-North West  R- 9-12 Moderate  S-0-50  T-Peer reviewed journal article

**87 Eaton et al 2014.docx**

**Groups:**

2014  A-Impact of HIV and AIDS and/or links with food security  L-Urban  M-HFIAS  P-Western Cape  R- 13-15 Good  S->500  T-Peer reviewed journal article

**88 Crush and Caeser 2014**

**Groups:**

2014  A-Agriculture, agribusiness and agricultural extension  A-Water security and food security  L-Urban  M-HFIAS  M-Household Dietary Diversity 24 hour recall  P-KwaZulu Natal  R- 13-15 Good  S->500  T-Peer reviewed journal article

**89 D'Haese et al 2015.pdf**

**Groups:**

2014  A-Impact Assessment or Feasibility Study  L-Rural  M-HFIAS  M-Household Dietary Diversity 24 hour recall  P-KwaZulu Natal  R- 13-15 Good  S-201-500  T-Peer reviewed journal article

**90 Misselhorn, 2009.pdf**

**Groups:**

2009  A-Links between social capital and food security  L-Peri-urban  M-Household Dietary Diversity 24 hour recall  P-KwaZulu Natal  R- 13-15 Good  S-51-100  T-Peer reviewed journal article

**91 Jones, 2011.pdf**

**Groups:**

2011  A-Impact of HIV and AIDS and/or links with food security  L-Urban  P-Eastern Cape  R- 9-12 Moderate  S-0-50  T-Peer reviewed journal article

**92 Maliwichi et al 2010.pdf**

**Groups:**

2010  A-Impact Assessment or Feasibility Study  L-Rural  P-Limpopo  R- 9-12 Moderate  S-51-100  T-Peer reviewed journal article

**93 kolahdooz et al 2013.pdf**

**Groups:**

2013  A-State of food insecurity and/or nutrition  L-Rural  M-Household Dietary Diversity 24 hour recall  P-KwaZulu Natal  R- 13-15 Good  S-101-200  T-Peer reviewed journal article

**94 Le roux et al 2010.pdf**

**Groups:**

2010  A-Impact Assessment or Feasibility Study  L-Urban  M-Anthropometric measures  P-Western Cape  R - 16-18 Excellent  S->500  T-Peer reviewed journal article

**95 Madhaven & Townsend 2007.pdf**

**Groups:**

2007  A-Causes of food insecurity  A-State of food insecurity and/or nutrition  L-Rural  M-Anthropometric measures  P-Limpopo  R - 16-18 Excellent  S-201-500  T-Peer reviewed journal article

**96 Saloojee et al 2007.pdf**

**Groups:**

2007  A-Causes of food insecurity  L-Rural  M-Anthropometric measures  M-Household dietary diversity 7 day recall  M-Malnutrition disorders or disease  P-Limpopo  R - 16-18 Excellent  S-201-500  T-Peer reviewed journal article

**97 Van der hoeven et al 2013.pdf**

**Groups:**

2013  A-Role of traditional green leafy vegetables in food security  A-Wild foods, traditional indigenous food, indigenous knowledge  L-Rural  P-North West  R - 16-18 Excellent  S-51-100  T-Peer reviewed journal article

**98 Mamabola et al 2005.pdf**

**Groups:**

2005  A-State of food insecurity and/or nutrition  L-Rural  M-Anthropometric measures  P-Limpopo  R - 16-18 Excellent  S-101-200  T-Peer reviewed journal article

**99 Phometsi et al 2006.pdf**

**Groups:**

2006  A-Causes of food insecurity  A-Nutrition knowledge  L-Rural  M-Nutrition knowledge  P-North West  R- 9-12 Moderate  S-101-200  T-Peer reviewed journal article

**100 Faber et al 2005.pdf**

**Groups:**

2005  A-Assessing association between maternal and child nutrition status  L-Rural  M-Anthropometric measures  M-Blood tests  P-KwaZulu Natal  R- 13-15 Good  S-201-500  T-Peer reviewed journal article

**101 Shisanya and Hendriks 2011.pdf**

**Groups:**

2011  A-Community gardens and homestead gardens  A-Role of community or homestead gardens in food security  L-Rural  M-HFIAS  P-KwaZulu Natal  R- 13-15 Good  S-51-100  T-Peer reviewed journal article

**103 Faber et al 2013.pdf**

**Groups:**

2013  A-State of food insecurity and/or nutrition  L-Peri-urban  M-Household Dietary Diversity 24 hour recall  P-KwaZulu Natal  R - 16-18 Excellent  S-201-500  T-Peer reviewed journal article

**104 Kirsten et al 1998.pdf**

**Groups:**

1998  A-Contribution of production to food security  L-Rural  M-Anthropometric measures  P-KwaZulu Natal  R- 9-12 Moderate  S-101-200  T-Peer reviewed journal article

**105 Lemke 2015.pdf**

**Groups:**

2014  A-Causes of food insecurity  A-Explore farm worker food security situation  L-Rural  P-North West  R- 9-12 Moderate  S-not specified  T-Peer reviewed journal article

**106 Faber and Laubscher 2008.pdf**

**Groups:**

2008  A-State of food insecurity and/or nutrition  L-Rural  M-Food frequency questions  M-Household Dietary Diversity 24 hour recall  P-KwaZulu Natal  R- 9-12 Moderate  S-101-200  T-Peer reviewed journal article

**107 Taylor and Jinabhai 2001.pdf**

**Groups:**

2001  A-Impact Assessment or Feasibility Study  L-Rural  M-Not directly measured or measure unspecified  P-KwaZulu Natal  R- 13-15 Good  S-51-100  T-Peer reviewed journal article

**108 Musemwa et al 2015.pdf**

**Groups:**

2014  A-Causes of food insecurity  A-State of food insecurity and/or nutrition  L-Rural  M-HFIAS  P-Eastern Cape  R - 16-18 Excellent  S-101-200  T-Peer reviewed journal article

**109 Ruysenaar 2013.pdf**

**Groups:**

2012  A-Role of urban agriculture in livelihoods and food security  A-Urban food system  L-Urban  M-HFIAS  M-Household Dietary Diversity 24 hour recall  M-Months adequate food provisioning  P-Gauteng  R- 13-15 Good  S-101-200  T-Peer reviewed journal article

**110 Hunter et al 2007.pdf**

**Groups:**

2007  A-Household shocks and food security  A-Shocks, stressors, coping strategies  L-Rural  M-Not directly measured or measure unspecified  P-Mpumalanga  R - 16-18 Excellent  S-201-500  T-Peer reviewed journal article

**111 Cluver et al 2009.pdf**

**Groups:**

2009  A-Impact of HIV and AIDS and/or links with food security  L-Urban  M-Number of days with no food in last 7  P-Western Cape  R - 16-18 Excellent  S->500  T-Peer reviewed journal article

**112 Hope et al 2008.pdf**

**Groups:**

2008  A-Impact Assessment or Feasibility Study  A-Irrigation scheme and food security  L-Rural  M-Not directly measured or measure unspecified  P-Limpopo  R- 13-15 Good  S->500  T-Peer reviewed journal article

**113 Dovie et al 2007.pdf**

**Groups:**

2007  A-Wild foods  A-Wild foods, traditional indigenous food, indigenous knowledge  L-Rural  M-Not directly measured or measure unspecified  P-Limpopo  R- 13-15 Good  S-0-50  T-Peer reviewed journal article

**114 Steenkamp et al 2014.pdf**

**Groups:**

2014  A-Impact of HIV and AIDS and/or links with food security  L-Urban  P-Eastern Cape  R- 13-15 Good  S->500  T-Peer reviewed journal article

**115 Kruger et al 2006.pdf**

**Groups:**

2005  A-Explore farm worker food security situation  L-Rural  M-Anthropometric measures  P-North West  R- 9-12 Moderate  S-51-100  T-Peer reviewed journal article

**116 Patel and Hochfeld 2011.pdf**

**Groups:**

2011  A-Explore impact of social grant/s  L-Urban  M-Not directly measured or measure unspecified  P-Gauteng  R- 13-15 Good  S-201-500  T-Peer reviewed journal article

**117 Dewing et al 2013.pdf**

**Groups:**

2013  A-Association between food insecurity and post natal depression  L-Peri-urban  M-HFIAS  P-Western Cape  R - 16-18 Excellent  S-201-500  T-Peer reviewed journal article

**118 Swaans et al 2009.pdf**

**Groups:**

2009  A-Impact of HIV and AIDS and/or links with food security  L-Rural  P-KwaZulu Natal  R- 13-15 Good  S-0-50  T-Peer reviewed journal article

**119 DeSilva et al 2012.pdf**

**Groups:**

2012  A-Impact of HIV and AIDS and/or links with food security  L-Participants drawn from urban and rural  M-Selected questions from existing tools  P-KwaZulu Natal  R - 16-18 Excellent  S->500  T-Peer reviewed journal article

**120 Mitchell and Andersson 2011.pdf**

**Groups:**

2011  A-State of food insecurity and/or nutrition  L-Participants drawn from urban and rural  M-Not directly measured or measure unspecified  P-Eastern Cape  R- 13-15 Good  S->500  T-Peer reviewed journal article

**121 Cockburn et al 2014.pdf**

**Groups:**

2014  A-Agriculture, agribusiness and agricultural extension  A-Role of sugarcane farming in livelihoods  L-Rural  M-Not directly measured or measure unspecified  P-KwaZulu Natal  R- 9-12 Moderate  S-0-50  T-Peer reviewed journal article

**122 Drimie et al 2009.pdf**

**Groups:**

2009  A-Agriculture, agribusiness and agricultural extension  A-Role of agriculture in food security  L-Rural  M-Not directly measured or measure unspecified  P-Limpopo  R- 13-15 Good  S-not specified  T-Peer reviewed journal article

**123 Hart 2010.pdf**

**Groups:**

2010  A-Agriculture, agribusiness and agricultural extension  A-Potential for increased small-holder agricultural production to improve nutrition  L-Rural  M-Household hunger scale  P-Limpopo  R - 16-18 Excellent  S-101-200  T-Peer reviewed journal article

**124 Thandeka et al 2011.pdf**

**Groups:**

2011  A-Consumption / role of traditional and indigenous crops  A-Wild foods, traditional indigenous food, indigenous knowledge  L-Rural  M-Not directly measured or measure unspecified  P-KwaZulu Natal  R- 13-15 Good  S-51-100  T-Peer reviewed journal article

**125 Earl 2010 thesis.pdf**

**Groups:**

2010  A-Contribution of food gardens to food security  A-Role of community or homestead gardens in food security  L-Rural  M-Not directly measured or measure unspecified  P-Free State  R- 9-12 Moderate  S-51-100  T-Thesis or research report

**126 Urmilla, 2002.pdf**

**Groups:**

2002  A-Impact Assessment or Feasibility Study  L-Rural  M-Not directly measured or measure unspecified  P-KwaZulu Natal  R- 9-12 Moderate  S-0-50  T-Peer reviewed journal article

**127 Mekuria and Moletsane 1997.pdf**

**Groups:**

1997  A-State of food insecurity and/or nutrition  L-Rural  M-Food grown or production deficit  M-Self assessed food security  P-Northern  R- 13-15 Good  S-101-200  T-Peer reviewed journal article

**128 Kirsten et al 1996.pdf**

**Groups:**

1996  A-Causes of food insecurity  A-State of food insecurity and/or nutrition  L-Rural  M-Not directly measured or measure unspecified  P-KwaZulu Natal  R- 9-12 Moderate  S-101-200  T-Peer reviewed journal article

**129 Lorathu 2005 thesis.pdf**

**Groups:**

2005  A-Investigate challenges to food security interventions  L-Rural  M-Not directly measured or measure unspecified  P-North West  R - 0-8 Low  S-0-50  T-Thesis or research report

**130 Pappin et al 2015.pdf**

**Groups:**

2014  A-Links between orphanhood and food security  L-Urban  M-Number meals per day  M-Number of days with no food in last 7  P-Free State  R - 16-18 Excellent  S-201-500  T-Peer reviewed journal article

**131 Spearing et al 2013.pdf**

**Groups:**

2013  A-State of food insecurity and/or nutrition  L-Rural  M-Food quantiy estimates and nutrient content  M-Household Dietary Diversity 24 hour recall  P-KwaZulu Natal  R- 9-12 Moderate  S-51-100  T-Peer reviewed journal article

**132 Ogunlade et al 2011.pdf**

**Groups:**

2011  A-Impact Assessment or Feasibility Study  L-Urban  M-Anthropometric measures  M-Blood tests  M-Household Dietary Diversity 24 hour recall  P-North West  R - 16-18 Excellent  S-101-200  T-Peer reviewed journal article

**133 Khumalo et al 2011.pdf**

**Groups:**

2011  A-Consumer acceptability and perceptions of processed cereals including maize meal  L-Rural  M-Not directly measured or measure unspecified  P-Limpopo  R- 9-12 Moderate  S-0-50  T-Peer reviewed journal article

**134 de Villiers et al 2005.pdf**

**Groups:**

2005  A-Links between food security and primarly health care accessibility  L-Urban  M-Not directly measured or measure unspecified  P-Eastern Cape  R - 16-18 Excellent  S-0-50  T-Peer reviewed journal article

**135 McGarry and Shackleton 2009.pdf**

**Groups:**

2009  A-Wild foods  A-Wild foods, traditional indigenous food, indigenous knowledge  L-Rural  M-Household Dietary Diversity 24 hour recall  M-Individual dietary diversity index  P-Eastern Cape  R - 16-18 Excellent  S->500  T-Peer reviewed journal article

**136 Dannhauser et al 1999.pdf**

**Groups:**

1999  A-Impact of HIV and AIDS and/or links with food security  L-Participants drawn from urban and rural  M-Anthropometric measures  M-Food frequency questions  M-Household Dietary Diversity 24 hour recall  P-Free State  R- 13-15 Good  S-51-100  T-Peer reviewed journal article

**137 Faber and Benade 2001.pdf**

**Groups:**

2001  A-Consumer acceptability and perceptions of processed cereals including maize meal  L-Rural  M-Household Dietary Diversity 24 hour recall  P-KwaZulu Natal  R- 9-12 Moderate  S-51-100  T-Peer reviewed journal article

**138 de Villiers and Senekal 2002.pdf**

**Groups:**

2002  A-Causes of food insecurity  L-Urban  M-Anthropometric measures  M-Food frequency questions  P-Eastern Cape  R- 13-15 Good  S-101-200  T-Peer reviewed journal article

**139 Walker and walker 1997.pdf**

**Groups:**

1997  A-Causes of food insecurity  L-Rural  M-Anthropometric measures  M-Household Dietary Diversity 24 hour recall  P-North West  R- 9-12 Moderate  S-201-500  T-Peer reviewed journal article

**140 Reddy et al 2014.pdf**

**Groups:**

2014  A-Explore gender dimensions and determinants of food security  A-Women or gender and food security  L-Rural  M-Months adequate food provisioning  P-Limpopo  P-Mpumalanga  R- 13-15 Good  S-51-100  T-Technical or other unplushed report

**141 Abrahams et al 2010.pdf**

**Groups:**

2010  A-State of food insecurity and/or nutrition  L-Participants drawn from urban and rural  M-Anthropometric measures  M-Household Dietary Diversity 24 hour recall  P-Western Cape  R- 13-15 Good  S->500  T-Peer reviewed journal article

**142 Hart 2011.pdf**

**Groups:**

2011  A-Agriculture, agribusiness and agricultural extension  A-Role of extension services in food security  A-Role of traditional green leafy vegetables in food security  A-Wild foods, traditional indigenous food, indigenous knowledge  L-Rural  M-Not directly measured or measure unspecified  P-Limpopo  R - 16-18 Excellent  S-101-200  T-Peer reviewed journal article

**143 Aphane et al 2010.pdf**

**Groups:**

2010  A-Explore livelihood strategies and food security  L-Rural  M-Not directly measured or measure unspecified  P-Eastern Cape  P-Limpopo  R- 9-12 Moderate  S-201-500  T-Peer reviewed journal article

**144 DeCock et al 2013.pdf**

**Groups:**

2013  A-State of food insecurity and/or nutrition  L-Rural  M-Food Expenditure  M-HFIAS  M-Household dietary diversity 7 day recall  M-Months adequate food provisioning  P-Limpopo  R - 16-18 Excellent  S->500  T-Peer reviewed journal article

**145 Murugani et al 2014.pdf**

**Groups:**

2004  A-Explore gender dimensions and determinants of food security  A-Land rights  A-Women or gender and food security  L-Rural  M-Not directly measured or measure unspecified  P-Limpopo  R- 9-12 Moderate  S-101-200  T-Peer reviewed journal article

**146 Rasethe et al 2013.pdf**

**Groups:**

2013  A-Wild foods  A-Wild foods, traditional indigenous food, indigenous knowledge  L-Rural  M-Not directly measured or measure unspecified  P-Limpopo  R - 0-8 Low  S-51-100  T-Peer reviewed journal article

**147 Rampedi and Olivier 2013.pdf**

**Groups:**

2013  A-Wild foods  A-Wild foods, traditional indigenous food, indigenous knowledge  L-Rural  M-Not directly measured or measure unspecified  P-Limpopo  R - 0-8 Low  S-51-100  T-Peer reviewed journal article

**148 Mosina et al 2013.pdf**

**Groups:**

2013  A-Contribution of food gardens to food security  A-Role of community or homestead gardens in food security  A-Role of urban agriculture in livelihoods and food security  A-Urban food system  L-Peri-urban  M-Not directly measured or measure unspecified  P-Limpopo  R- 9-12 Moderate  S-51-100  T-Peer reviewed journal article

**149 Theron et al 2006.pdf**

**Groups:**

2006  A-Causes of food insecurity  L-Participants drawn from urban and rural  M-Anthropometric measures  M-Food frequency questions  M-Food quantiy estimates and nutrient content  P-Gauteng  P-Limpopo  R- 13-15 Good  S-101-200  T-Peer reviewed journal article

**150 Twine et al 2003.pdf**

**Groups:**

2003  A-Wild foods  A-Wild foods, traditional indigenous food, indigenous knowledge  L-Rural  M-Not directly measured or measure unspecified  P-Limpopo  R- 9-12 Moderate  S-101-200  T-Peer reviewed journal article

**151 Faber et al 2009.pdf**

**Groups:**

2009  A-Development of food security measurement tool/s  A-Dietary diversity and food security  L-Rural  M-Anthropometric measures  M-HFIAS  M-Household Dietary Diversity 24 hour recall  M-Months adequate food provisioning  P-Limpopo  R - 16-18 Excellent  S-201-500  T-Peer reviewed journal article

**152 Sharaunga et al 2015.pdf**

**Groups:**

2014  A-Explore gender dimensions and determinants of food security  A-Women or gender and food security  L-Rural  M-Not directly measured or measure unspecified  P-KwaZulu Natal  R- 13-15 Good  S-201-500  T-Peer reviewed journal article

**153 Modi et al 2006.pdf**

**Groups:**

2006  A-Wild foods  A-Wild foods, traditional indigenous food, indigenous knowledge  L-Rural  M-Not directly measured or measure unspecified  P-KwaZulu Natal  R- 9-12 Moderate  S-0-50  T-Peer reviewed journal article

**154 Zobolo and Mkabela 2006.pdf**

**Groups:**

2006  A-Role of indigenous knowledge  A-Wild foods, traditional indigenous food, indigenous knowledge  L-Rural  M-Not directly measured or measure unspecified  P-KwaZulu Natal  R - 0-8 Low  S-51-100  T-Peer reviewed journal article

**155 Kaschula 2008.pdf**

**Groups:**

2008  A-Impact of HIV and AIDS and/or links with food security  L-Rural  M-Coping strategy index  M-Household dietary diversity 48hour recall  M-Household hunger scale  P-KwaZulu Natal  R- 9-12 Moderate  S-101-200  T-Peer reviewed journal article

**156 Tee et al 2015.pdf**

**Groups:**

2014  A-State of food insecurity and/or nutrition  L-Participants drawn from urban and rural  M-Household Dietary Diversity 24 hour recall  P-North West  R- 13-15 Good  S-201-500  T-Peer reviewed journal article

**157 Cross and Altman 2010.pdf**

**Groups:**

2010  A-Explore gender dimensions and determinants of food security  A-Women or gender and food security  L-Participants drawn from urban and rural  M-Self assessed food security  P-Gauteng  P-Mpumalanga  R- 9-12 Moderate  S-0-50  T-Peer reviewed journal article

**158 Trefry et al 2014.pdf**

**Groups:**

2014  A-Culture and food security  L-Rural  M-Not directly measured or measure unspecified  P-Eastern Cape  R- 13-15 Good  S-0-50  T-Peer reviewed journal article

**159 Arko-Achemfuor 2014.pdf**

**Groups:**

2014  A-Impact Assessment or Feasibility Study  L-Rural  M-Not directly measured or measure unspecified  P-North West  R - 0-8 Low  S-0-50  T-Peer reviewed journal article

**160 Nkosi 2005 thesis.pdf**

**Groups:**

2005  A-State of food insecurity and/or nutrition  L-Rural  M-Household hunger scale  P-KwaZulu Natal  R- 13-15 Good  S-51-100  T-Thesis or research report

**161 Hooper 2004 MSc report.pdf**

**Groups:**

2004  A-Agriculture, agribusiness and agricultural extension  A-Role of extension services in food security  L-Rural  M-Not directly measured or measure unspecified  P-Western Cape  R - 0-8 Low  S-0-50  T-Thesis or research report

**162 deSilva 2011 Masters.pdf**

**Groups:**

2011  L-Peri-urban  M-Not directly measured or measure unspecified  P-KwaZulu Natal  R - 0-8 Low  S-0-50  T-Thesis or research report

**163 Letts 2013 Thesis.pdf**

**Groups:**

2013  A-Role of urban agriculture in livelihoods and food security  A-Urban food system  L-Urban  M-HFIAS  M-Months adequate food provisioning  P-Western Cape  R- 13-15 Good  S-51-100  T-Thesis or research report

**164 Rajput 2012 thesis.pdf**

**Groups:**

2012  A-Impact Assessment or Feasibility Study  L-Rural  M-Not directly measured or measure unspecified  P-Free State  R- 13-15 Good  S-0-50  T-Thesis or research report

**165 Gumede 2013 thesis.pdf**

**Groups:**

2013  A-State of food insecurity and/or nutrition  L-Rural  M-Not directly measured or measure unspecified  P-KwaZulu Natal  R - 0-8 Low  S-0-50  T-Thesis or research report

**166 Lunga 2011 thesis.pdf**

**Groups:**

2010  A-Impact Assessment or Feasibility Study  L-Rural  P-KwaZulu Natal  R - 0-8 Low  S-0-50  T-Thesis or research report

**167 Dlamini_2005 thesis.pdf**

**Groups:**

2005  A-Impact Assessment or Feasibility Study  L-Rural  M-Not directly measured or measure unspecified  P-KwaZulu Natal  R- 9-12 Moderate  S-0-50  T-Thesis or research report

**168 Mfundo 2013 thesis.pdf**

**Groups:**

2013  A-Infrastructure and food security  L-Rural  M-Not directly measured or measure unspecified  P-KwaZulu Natal  R- 9-12 Moderate  S-51-100  T-Thesis or research report

**169 Oppong_2013 thesis.pdf**

**Groups:**

2013  A-Wild foods  A-Wild foods, traditional indigenous food, indigenous knowledge  L-Rural  M-HFIAS  P-Limpopo  R- 13-15 Good  S-101-200  T-Thesis or research report
