# Supplementary figures and images for: A systematic review of sub-national food insecurity research in South Africa: Missed opportunities for policy insights
Source: PLoS One. 2017 Aug 22;12(8):e0182399. doi: 10.1371/journal.pone.0182399 (PMC5567909; doi:10.1371/journal.pone.0182399)

**S2 Annex of terms**

**S2:1 Levels of food insecurity**


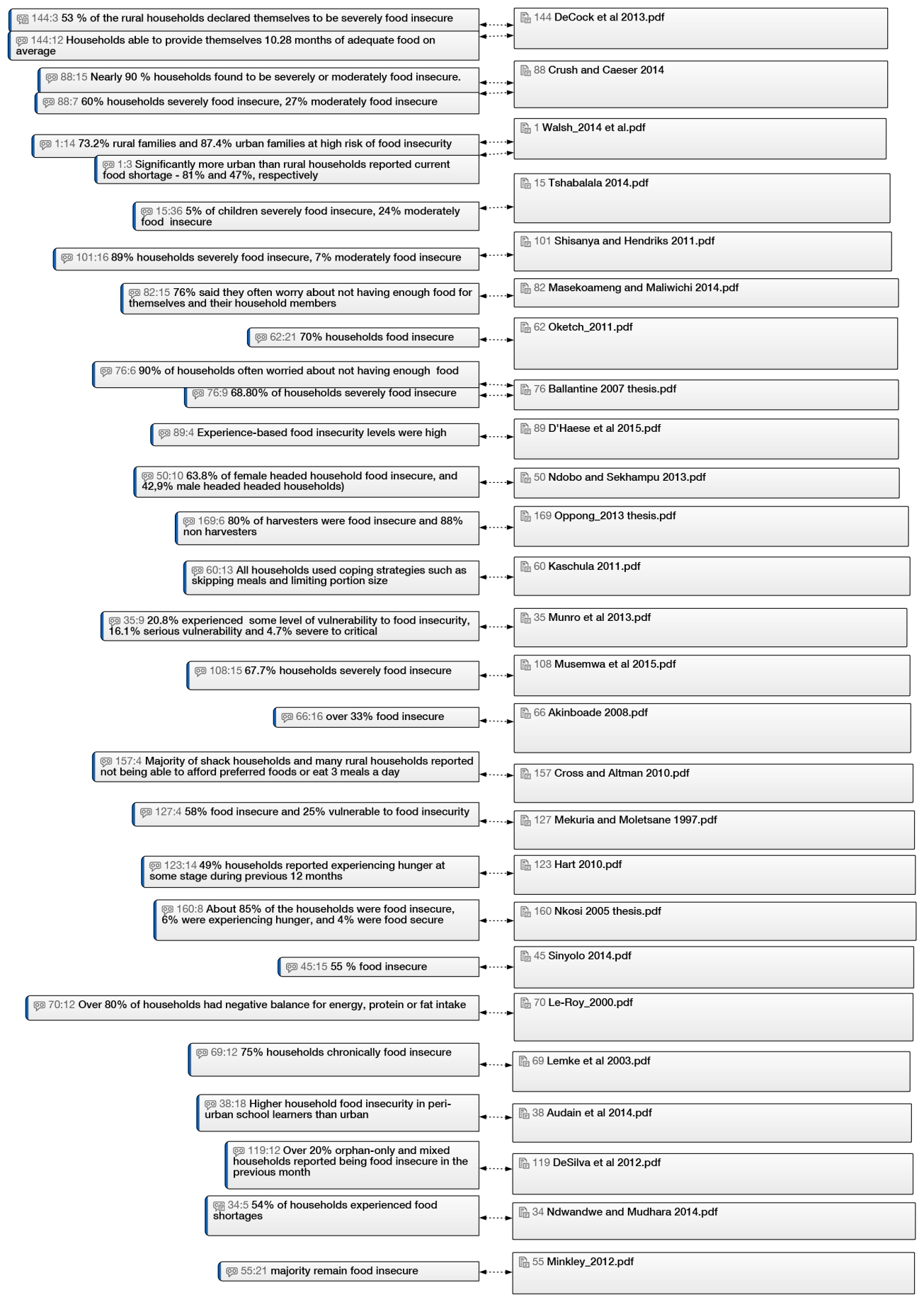


S2:2 Diet quality


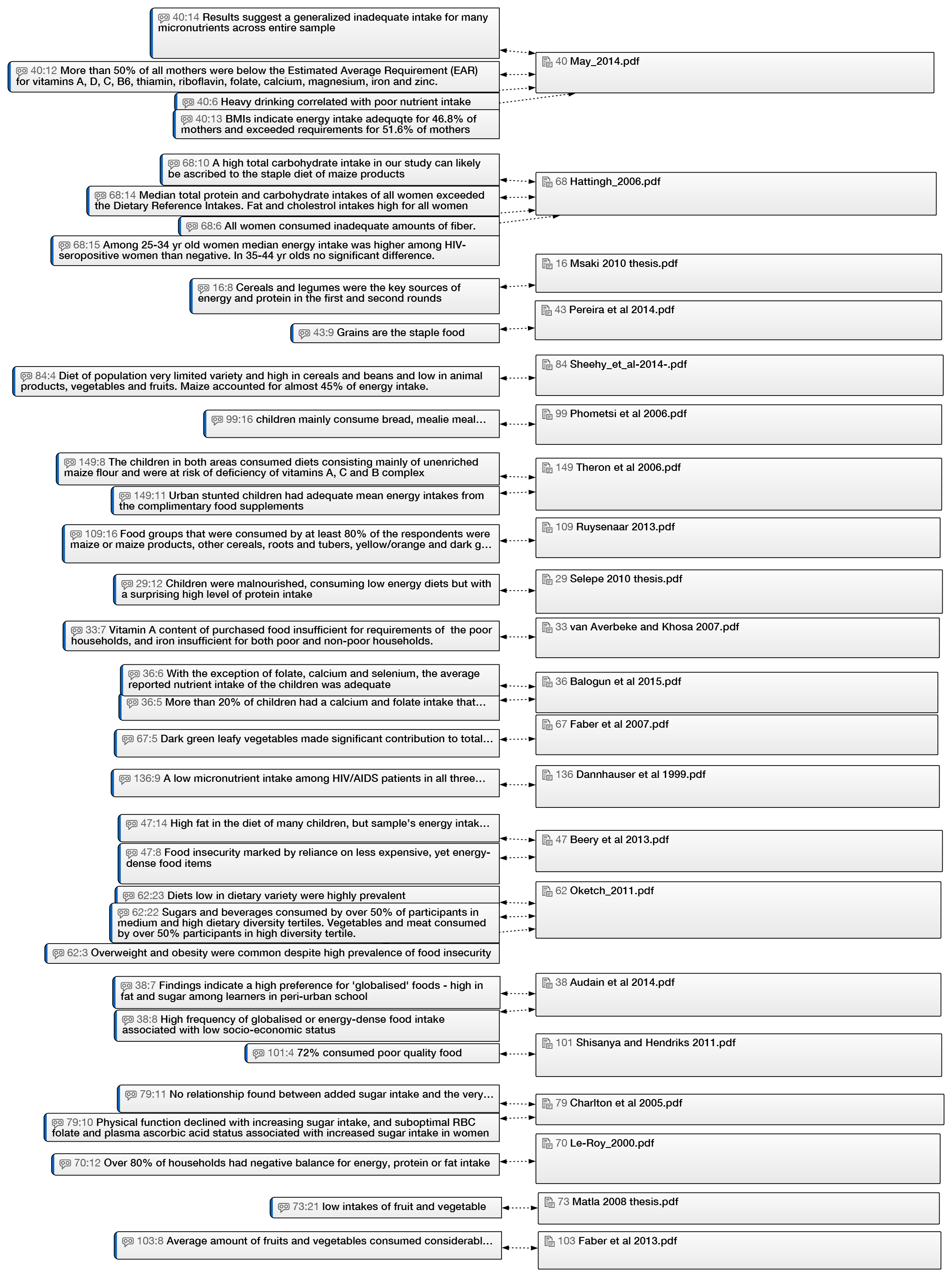


S2:3 Anthropometry


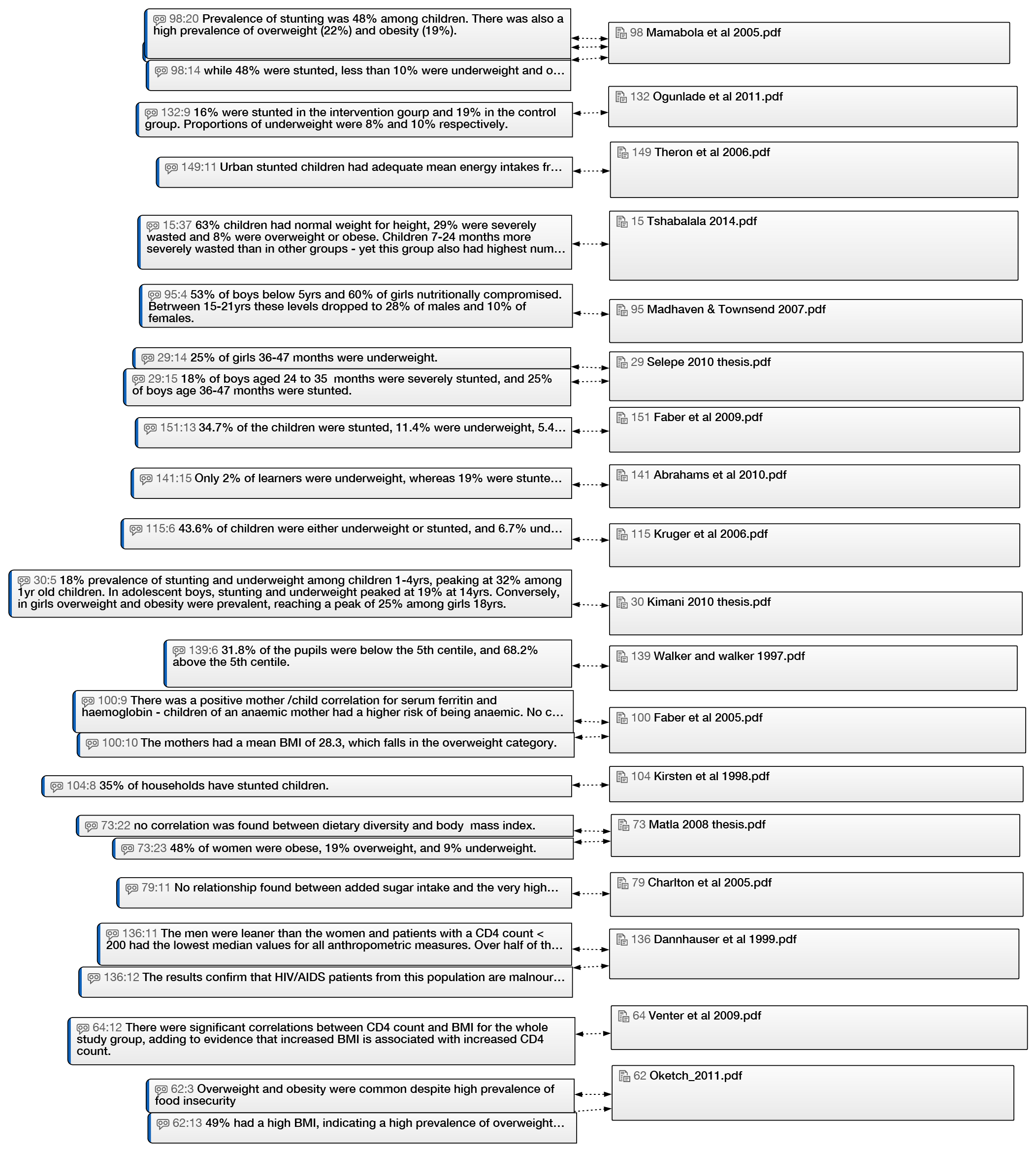

Supplement: S1 File — (DOCX) [file pone.0182399.s002.docx]
